# Serum Anti-14-3-3 Zeta Autoantibody as a Biomarker for Predicting Hepatocarcinogenesis

**DOI:** 10.3389/fonc.2021.733680

**Published:** 2021-10-15

**Authors:** Ting Wang, Xue-ying Huang, Su-jun Zheng, Ye-ying Liu, Si-si Chen, Feng Ren, Jun Lu, Zhong-ping Duan, Mei Liu

**Affiliations:** ^1^ Department of Respiratory and Infectious Diseases, Beijing You’an Hospital, Capital Medical University, Beijing, China; ^2^ Department of Oncology, Beijing You’an Hospital, Capital Medical University, Beijing, China; ^3^ First Department of Hepatology Center, Beijing You’an Hospital, Capital Medical University, Beijing, China; ^4^ Beijing Institute of Hepatology, Beijing You’an Hospital, Capital Medical University, Beijing, China; ^5^ Fourth Department of Hepatology Center, Beijing You’an Hospital, Capital Medical University, Beijing, China

**Keywords:** SWATH-MS proteome technology, 14-3-3 zeta, biomarker, hepatocarcinogenesis, premalignant liver disease

## Abstract

Hepatocellular carcinoma (HCC) is a common malignancy worldwide. Alpha-fetoprotein (AFP) is still the only serum biomarker widely used in clinical settings. However, approximately 40% of HCC patients exhibit normal AFP levels, including very early HCC and AFP-negative HCC; for these patients, serum AFP is not applicable as a biomarker of early detection. Thus, there is an urgent need to identify novel biomarkers for patients for whom disease cannot be diagnosed early. In this study, we screened and identified novel proteins in AFP-negative HCC and evaluated the feasibility of using autoantibodies to those protein to predict hepatocarcinogenesis. First, we screened and identified differentially expressed proteins between AFP-negative HCC tissue and adjacent non-tumor liver tissue using SWATH-MS proteome technology. In total, 2,506 proteins were identified with a global false discovery rate of 1%, of which 592 proteins were expressed differentially with 175 upregulated and 417 downregulated (adjusted *p*-value <0.05, fold-change FC ≥1.5 or ≤0.67) between the tumor and matched benign samples, including 14-3-3 zeta protein. For further serological verification, autoantibodies against 14-3-3 zeta in serum were evaluated using enzyme-linked immunosorbent, Western blotting, and indirect immunofluorescence assays. Five serial serum samples from one patient with AFP-negative HCC showed anti-14-3-3 zeta autoantibody in sera 9 months before the diagnosis of HCC, which gradually increased with an increase in the size of the nodule. Based on these findings, we detected the prevalence of serum anti-14-3-3 zeta autoantibody in liver cirrhosis (LC) patients, which is commonly considered a premalignant liver disease of HCC. We found that the prevalence of autoantibodies against 14-3-3 zeta protein was 16.1% (15/93) in LC patient sera, which was significantly higher than that in patients with chronic hepatitis (0/75, *p* = 0.000) and normal human sera (1/60, 1.7%, *p* = 0.01). Therefore, we suggest that anti-14-3-3 zeta autoantibody might be a biomarker for predicting hepatocarcinogenesis. Further follow-up and research of patients with positive autoantibodies will be continued to confirm the relationship between anti-14-3-3 zeta autoantibody and hepatocarcinogenesis.

## 1 Introduction

Hepatocellular carcinoma (HCC) is a common malignancy worldwide, with approximately 840,000 new cases annually. Nearly 800,000 people die of HCC every year (1). Alpha-fetoprotein (AFP) is still the only serum biomarker widely used clinically. However, approximately 40% of cases with normal AFP levels, including those with very early HCC and AFP-negative HCC, cannot be detected early. Thus, there is an urgent need to identify novel biomarkers for patients for whom disease cannot be diagnosed early. Liver cirrhosis (LC) is a common chronic progressive liver disease caused by long-term or repeated diffuse liver damage due to one or more causes (2). It is known that underlying LC and chronic hepatitis (CH) are common precursors of HCC, and 80%–90% of HCCs develop from LC. Thus, LC is considered a premalignant disease of HCC (3). Therefore, autoantibodies detected in premalignant diseases of HCC, such as LC, are of significance for the identification of individuals who will potentially develop HCC.

Many studies have shown that the sera from cancer patients contain autoantibodies that react with a unique group of autologous cellular antigens known as tumor-associated antigens (TAAs) (4). These antigens are usually overexpressed, aberrantly cleaved, posttranslationally modified, mutated, or aberrantly localized in cancerous cells (5, 6). Autoantibodies to TAAs are sometimes detected during the transition to malignancy, such as from chronic liver disease to HCC (7). It has been proposed that these antibody responses could be stimulated by abnormal cellular proteins that are involved in carcinogenesis. Thus, the appearance of autoantibodies to TAAs might predict hepatocarcinogenesis. At present, there are few studies on tumor-associated antigens and autoantibodies to predict hepatocarcinogenesis, and no biomarkers with better sensitivity and specificity have been detected.

In this study, we aimed to screen and identify differentially expressed proteins in very early HCC and AFP-negative HCC and evaluate the feasibility of using autoantibodies against these identified antigens to predict hepatocarcinogenesis with premalignant liver disease, such as LC. We screened and identified novel proteins in AFP-negative HCC tissue that were differentially expressed related to levels in adjacent non-tumor liver tissue using SWATH-MS proteome technology and investigated the prevalence of serum autoantibodies against identified antigens in serial serum samples of AFP-negative HCC patients. Moreover, we sought to detect the prevalence of autoantibodies against identified proteins in LC patient sera and evaluate the feasibility of using serum autoantibodies to predict hepatocarcinogenesis.

## 2 Materials and Methods

### 2.1 Patients and Serum Samples

HCC tissues and adjacent non-tumorous liver tissue counterparts used for this study were collected from HBV-associated HCC patients who underwent hepatectomy. Sera from 93 patients with LC, 75 patients with CH, and 60 normal human serum (NHS) samples were obtained from outpatients or inpatients. Tissues and sera were collected at Beijing You’an Hospital, Capital Medical University, Beijing, China, between January 2015 and January 2016. HCC tissues and adjacent non-tumorous liver tissues were verified by histopathological examination. The diagnosis of LC and CH was based on clinical, biochemical, and imaging studies, and/or liver histological data, according to the Chronic Hepatitis B Prevention and Treatment Guidelines of 2015. One AFP-negative HCC patient was diagnosed according to the Primary Liver Cancer Treatment Protocols (2011 edition). Patients with LC and CH were followed up for at least 18 months to exclude individuals with primary biliary cirrhosis. This study was approved by the Institutional Review Board of Capital Medical University, Beijing, China (February 28, 2014). Patients/participants provided written informed consent to participate in the study.

### 2.2 Recombinant Proteins and Antibody

The 14-3-3 zeta construct GST-14-3-3 WT (plasmid ID: 1944), purchased from Addgene (75 Sidney Street, Suite 550A Cambridge, MA 02139, USA), was subcloned into the pET28a vector. The recombinant protein 14-3-3 zeta expressed in ArcticExpress (DE3) RP was purified using nickel column chromatography. Recombinant protein was examined using SDS-PAGE, and the expected molecular size of the expression products was determined using Coomassie blue staining. Western blot analysis was performed to confirm that the bands observed in SDS-PAGE were reactive with the corresponding antibodies.

### 2.3 Differential Proteomics Between AFP-Negative HCC and Adjacent Non-Tumor Liver Tissue

#### 2.3.1 Sample Preparation

The AFP-negative HCC tissues [AFP (−) -HCC group] and adjacent non-tumorous liver tissues (control group) were cut into pieces and washed with ice-cold phosphate-buffered saline (PBS) at 4°C. The samples were ground into powder in liquid nitrogen and then homogenized on ice in 1 ml of lysis buffer (50 mM HEPES, 6 M urea, 2 M thiourea, and 1× protease inhibitor cocktail). After that, the samples were vortexed for 10 s at 200 rpm 20 times following centrifugation at 20,000*×g* for 40 min at 4°C to collect the supernatant (total protein solution of each sample). The protein concentration of each sample was measured using the BCA protein assay method (8), and each group was subjected to biological triplicate experiments.

#### 2.3.2 Protein Digestion in Solution

Tissue protein solution (100 μg protein) was diluted with an equal volume of 20% 2,4,6-trichloroanisaole (TCA) to purify proteins. The solution was then precipitated for 2 h at −80°C and centrifuged at 20,000*×g* for 30 min at 4°C. The precipitate was suspended in 0.4 ml of acetone (4°C) *via* ultrasonic treatment for 5 min. The resulting mixture was continuously precipitated overnight at −80°C and centrifuged at 20,000*×g* for 30 min at 4°C, the acetone was removed, and the purified proteins were obtained.

Purified proteins were dissolved in 70 µl 0.2% RapiGest SF in 50 mM ABC solution and then reduced and alkylated by incubation with 100 mM tris (2-carboxyethyl) phosphine in 50 mM ABC solution for 30 min at 60°C and 100 mM iodoacetamide in 50 mM ABC solution for 30 min at room temperature in the dark, respectively. Digestion was performed by incubation with trypsin (Promega; enzyme-to-substrate ratio, 1:100) at 37°C with 160 rpm shaking overnight. The enzymatic reaction was stopped by adding 10% trifluoroacetic acid solution at a final concentration of 1% at 37°C for 0.5 h. The digestion was transferred to an ultrafiltration tube and centrifuged at 10,000*×g* for 20 min at 4°C, and the filtrate comprised the sample peptide solution at a final concentration of 1 μg/μl.

#### 2.3.3 Proteomic Data Acquisition

Identified proteomic data were acquired on an AB 5600+ Triple TOF mass spectrometer (AB Sciex) equipped with an Eksigent 400 nano-HPLC system (Eksigent, AB Sciex, USA). A nano-LC MS/MS method was built, and the main steps were as follows: an equal volume of each peptide solution was taken to obtain one pooled peptide solution. The mixed solution was loaded on a nano trap column (350 μm × 0.5 mm, Chrom XP C18-3 μm, 120 A) and washed with acetonitrile–water–formic acid (2:98:0.1, v/v/v) solution for desalting at a flow rate of 2 μl/min for 10 min. After desalting, peptides were separated on an analytical column (75 μm × 20 cm, Sunchrom C18-5 μm, 120, Beijing Happy Science Scientific Co., Ltd.) at a flow rate of 300 nl/min, and gradient elution was performed according to the following method [preparation of phase A (ACN–H_2_O–FA (98:2:0.1, v/v/v)] and phase B [ACN–H_2_O–FA (0.1:98:2, v/v/v)]: 0 min, 95% and 5%; 1 min, 91% and 9%; 95 min, 75% and 25%; 100 min, 50% and 50%; 101 min, 20% and 80%; 110 min, 20% and 80%; 111 min, 95% and 5%; 120 min, 95% and 5% phase B. The peptide elution was ionized, and then, MS data were acquired using a data-dependent acquisition (DDA) model with an AB 5600+ Triple TOF mass spectrometer.

In the high-resolution model, the first-order spectra were scanned in the range of 300–605 *m*/*z*, 600–805 *m*/*z*, and 800–1,250 *m*/*z*. The TOF MS parameters were set as follows: spray mode, nano-electrospray; GS1, 12; CUR, 30; ISDF, 2,500; IHF, 150; DP, 100; CE, 10; cumulative time, 250 ms; mass deviation, 50 mDa. The dynamic exclusion acquisition time was set to 20 s. The charge-setting range was 2–5. Fragment ion level 2 spectra (MS2) were acquired in high sensitivity mode, with a mass-to-charge ratio (*m*/*z*) ranging from 100 to 1,500 and an acquisition time of 250 ms per cycle.

SWATH label-free was introduced to quantify identified proteins, and the SWATH MS/MS method was established. At first, pooled peptide spectra were acquired with the nano-LC MS/MS method based on the DDA model, but the TOF MS range was set from *m*/*z* 300 to 1,250. The variable window for SWATH-MS was calculated using Variable Window Calculator V 0.2 112513 (AB Sciex). The parameters were set as follows: number of windows, 60; lower *m*/*z*, 300; upper *m*/*z*, 1,250; window overlap (Da), 0.5; rolling collision energy, 15. Product ion spectra were measured in the range of *m*/*z* 100 to 1,500 for 40 ms with the high sensitivity model, and the resulting total cycle time was 1.42 s. SWATH-MS measurements were performed in triplicate for each sample.

#### 2.3.4 Data Processing and Bioinformatics Analysis

DDA data were analyzed with Paragon (ProteinPilot software, AB/Sciex) against the *Homo sapiens* database (https://www.ncbi.nlm.nih.gov/genome/?term=Homo+sapiens). The detected protein threshold [unused Protscope (conf)] of result quality was 0.05 (10%), and false discovery rate (FDR) analysis was chosen. SWATH data were analyzed using PeakView SWATH Processing Micro App (AB Sciex), followed by real-time global correction. The important parameters for processing SWATH data were as follows: peptide confidence threshold, 95%; FDR threshold, 1.0%; 50 ppm *m*/*z* tolerance; 15 min extraction window; shared peptides excluded for SWATH analysis. Protein peak area data were finally obtained.

Protein areas were normalized, and principal component analysis (PCA) and *t*-tests were performed for AFP(−)-HCC *versus* CK. Differentially expressed proteins (DEPs) were considered based on a fold-change of ≥1.5 or ≤0.067 and a *p*-value less than 0.05. Next, DEPs were annotated based on the Gene Ontology Consortium (GO) (9), including cellular component, biological process, and molecular functions, and their systematic information was computed based on Kyoto Encyclopedia of Genes and Genomes (KEGG) annotation (10).

### 2.4 Enzyme-Linked Immunosorbent Assay

The 14-3-3 zeta recombinant protein was diluted in PBS to a final concentration of 0.5 µg/ml and used to coat a 96-well microtiter plate (Corning 3590, USA), which was incubated at 4°C overnight. The antigen-coated wells were blocked with 10% fetal bovine serum at 37°C for 1 h. Human serum (diluted 1:200) was incubated in the antigen-coated wells for 60 min. Horseradish peroxidase (HRP)-conjugated goat anti-human IgG (Beijing Zhong Shan-Golden Bridge Biological Technology Co., Ltd., China) as a secondary antibody was diluted to 1:10,000 for coating (60 min). Then, it was washed with PBS containing 0.1% Tween 20. A 3,3,5,5-tetramethylbenzidine liquid substrate system (Beijing Solarbio Science & Technology Co., Ltd., China) was used as the detection agent. The optical density (OD) value of all wells was 450 nm. The cutoff value for defining a positive reaction was designated as the mean OD value of the 60 NHS samples plus three standard deviations (mean + 3 SD). Each microtiter plate included 10 NHS samples representing a range of absorbance above and below the mean of 60 NHS samples. The average OD value of 10 NHS samples was used to normalize all OD values to the standard mean of the 60 normal samples. Each sample was tested in triplicate.

### 2.5 Western Blotting

The purified 14-3-3 zeta recombinant protein was electrophoresed on a 12% SDS-PAGE gel and subsequently transferred to a nitrocellulose membrane. After blocking in TBS with 5% non-fat milk and 0.1% Tween-20 for 1 h at ambient temperature (25°C), the membrane was cut into strips and incubated with patient sera diluted at 1:200 or polyclonal anti-14-3-3 zeta antibody diluted at 1:500. The membranes were finally incubated with HRP-conjugated goat anti-human IgG or HRP-conjugated goat anti-rabbit IgG diluted at 1:10,000 for 1 h. Positive signals were detected using an ECL kit (Thermo Scientific, Waltham, MA, USA) according to the instructions of the manufacturer.

### 2.6 Indirect Immunofluorescence Assay

An indirect immunofluorescence assay was performed on a Hep-2 cell substrate slide (Medical and Biological Laboratories Co., Ltd.). The sera were diluted to 1:40 with PBS at pH 7.4 and incubated with the slides for 30 min at ambient temperature, followed by extensive washing with PBS. The slides were then incubated with fluorescein isothiocyanate-conjugated goat anti-human IgG secondary antibody for 20 min at ambient temperature and thoroughly washed with PBS. Subsequently, a drop of mounting medium containing 4,6-diamidino-2-phenylindole (Beijing Zhong Shan-Golden Bridge Biological Technology Co., Ltd., China) was added. The slides were examined under a fluorescence microscope in the dark using a Nikon ECLIPSE Ti at ×200 magnification. Related software was used for image capture and analysis.

Continuous variables are expressed as the mean ± SD or median (25th and 75th percentiles). Count data were described as frequencies. The mean OD value in each group of patient sera was compared using the Mann–Whitney *U* test or one-way analysis of variance. The frequency of anti-14-3-3 zeta autoantibody in each group of patient sera was compared using the chi-squared (*χ* ^2^) test with Yate’s correction. Two significant levels (0.05, 0.01) were used.

## 3 Results

### 3.1 Differentially Expressed Proteomics of *In Situ* AFP-Negative HCC and Adjacent Non-Tumor Liver Tissue

**Figure 1:**
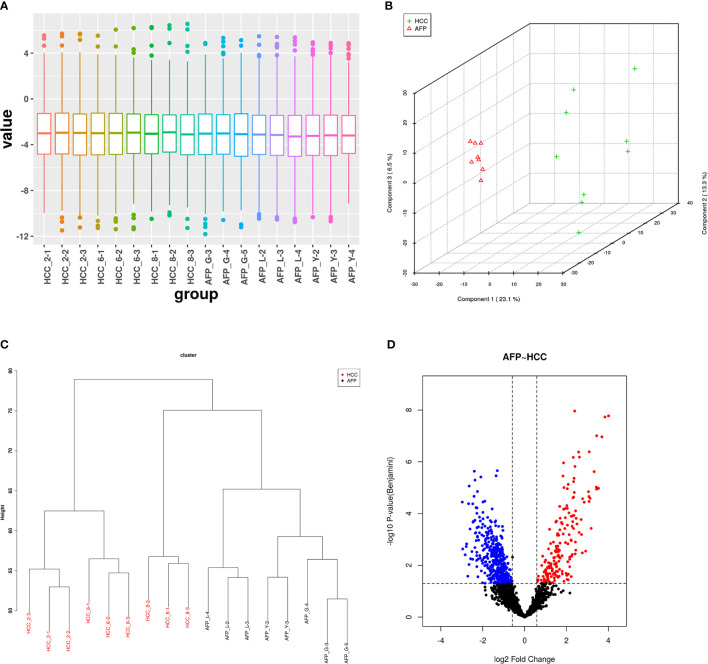
Quality control of SWATH quantification areas between the alpha-fetoprotein (AFP)-negative hepatocellular carcinoma (HCC) tissue and adjacent tissues. **(A)** Statistical results of normalized areas. There was significant specificity between the AFP-negative HCC tissue and adjacent tissues by principal component analysis (PCA) and clustering analysis **(B, C)**. **(D)** Differentially expressed proteins between the HCC tissue and adjacent tissues.

In this project, we identified protein expression *in situ* using AFP-negative HCC tissue [AFP(−)-HCC] and adjacent non-tumor liver tissue (control group, CK). In total, 2,506 proteins were identified with a global FDR of 1% from 17,645 distinct peptides. Then, those identified proteins were quantified by the SWATH label-free method, and quality control was performed. After normalizing the protein areas (**Figure 1A**), *t*-test and clustering analysis results showed good tissue specificity (**Figures 1B, C** **)**. In addition, there were 592 DEPs between AFP(−)-HCC and CK with 175 upregulated and 417 downregulated (adjusted *p*-value <0.05, fold-change ≥1.5 or 0.67; **Figure 1D**).

**Figure 2:**
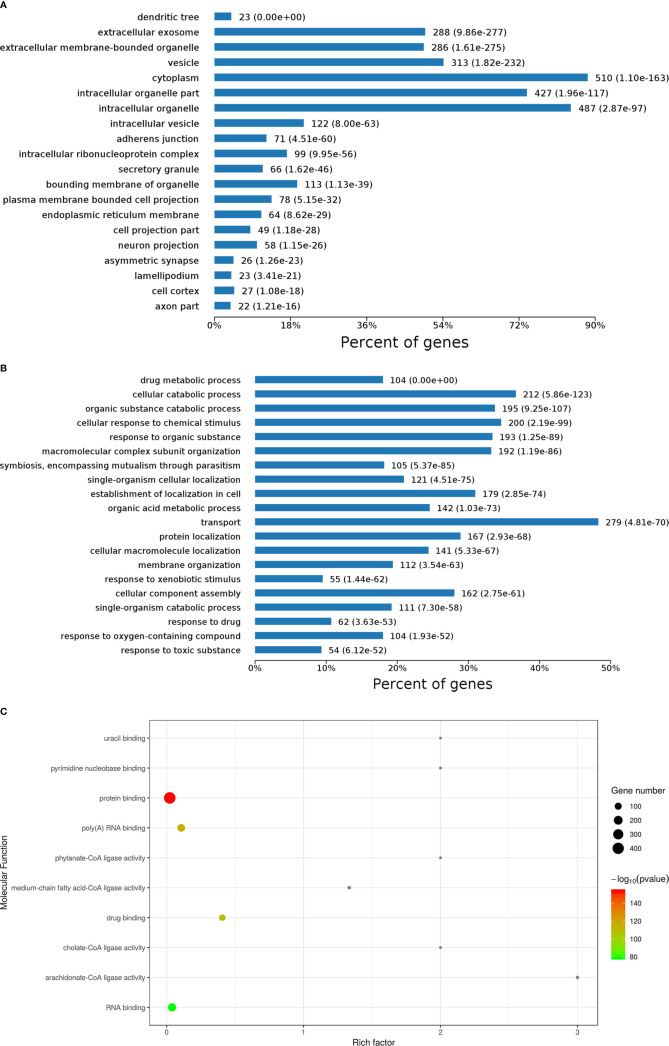
Gene ontology (GO) analysis results of differentially expressed proteins (DEPs) including cellular component **(A)**, biological process **(B)**, and molecular function **(C)** analysis and enrichment.

Cellular component results showed diverse locations of DEPs (**Figure 2A**). Specifically, 510 DEPs primarily located in the cytoplasm, followed by 487 DEPs located in intracellular organelles. Furthermore, 427 DEPs located in the intracellular organelle part. Moreover, 313 DEPs located in vesicles, 288 DEPs located in extracellular exosomes, and 286 DEPs located in extracellular membrane-bounded organelles. A few other DEPs located in intracellular vesicles, bounding membrane of organelles, and plasma membrane bounded cell projections, among others.

Biological processes of DEPs were also analyzed, as shown in **Figure 2B**. Here, 279 DEPs primarily involved in transport, followed by 212 DEPs involved in cellular catabolic processes, 200 DEPs involved in cellular response to chemical stimulus, 195 DEPs involved in organic substance catabolic processes, 193 DEPs involved in response to organic substances, 192 DEPs involved in molecular complex subunit organization, 179 DEPs involved in establishment of localization in cells, 167 DEPs involved in protein localization, and 162 DEPs involved in cellular component assembly. A few other DEPs involved in organic acid metabolic processes, cellular macromolecule localization, and single-organism cellular localization, among others.

In molecular function enrichment results (**Figure 2C**), binding and ligase activity were the predominant pathways. Ligase activity pathways comprised cholate-CoA ligase activity, arachidonate-CoA ligase activity, phytanate-CoA ligase activity, and medium-chain fatty acid-CoA ligase activity, with high rich factors but few DEPs. Binding was the major function of DEPs between AFP(−)-HCC and CK, including uracil binding, pyrimidine nucleobase binding, protein binding, poly (A) RNA binding, binding, RNA binding, and especially protein binding, which was associated with a significant *p*-value. 14-3-3 zeta (H0YB80) was one of those DEPs, and it performs protein domain-specific binding. It was reported that 14-3-3 zeta is a signaling molecule with discrete phosphoserine/threonine-binding modules that regulate tumor progression (11). Our previous studies also demonstrated that the sensitivity and specificity of immunologic diagnosis could be up to 69.7% and 83.0%, respectively, with 14 TAAs based on a microarray that included 14-3-3 zeta (12). Therefore, 14-3-3 zeta was considered a candidate TAA for AFP-negative HCC tests.

**Figure 3:**
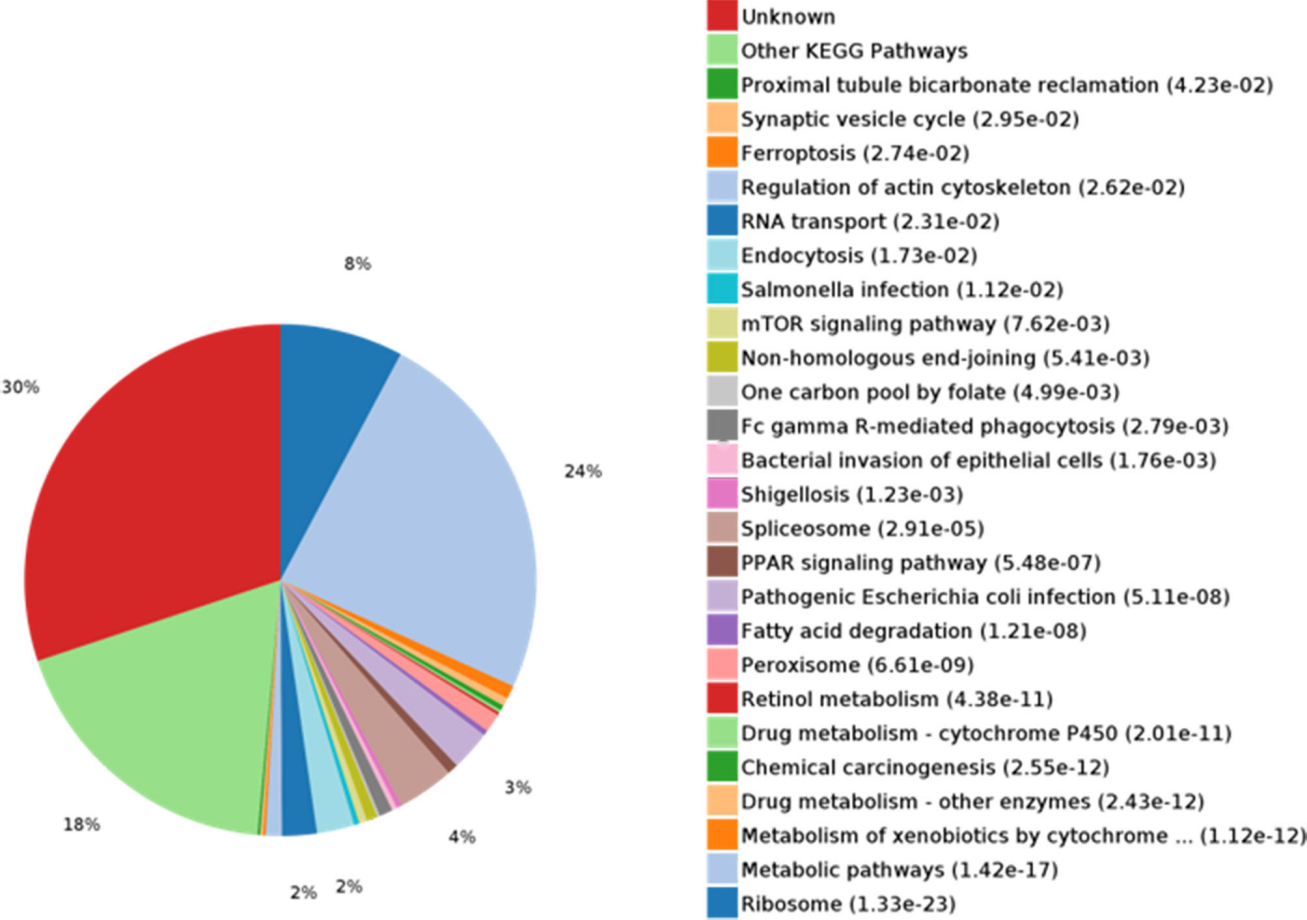
KEGG pathway analysis and enrichment of differentially expressed proteins (DEPs). KEGG metabolic pathway analysis showed that the metabolic pathways were rich in proteins (24%).

KEGG pathways for DEPs were analyzed and enriched (**Figure 3**). Metabolic pathways and ribosome were the most enriched pathways, followed by the spliceosome, pathogenesis, *Escherichia coli* infection, endocytosis, RNA transport, the mTOR signaling pathway, and PPAR signaling, among others. Signaling pathway molecules generally undergo phosphorylation. 14-3-3 proteins, as bridging molecules, can bind to phosphorylated protein molecules to transmit signals (13). If the phosphorylation of pathway molecules is inhibited, the binding fails and signal transmission is blocked. Therefore, 14-3-3 proteins play an important role in signal transduction and could be used as potential TAA-associated molecules.

### 3.2 Serological Verification in Serial Serum Samples

**Figure 4:**
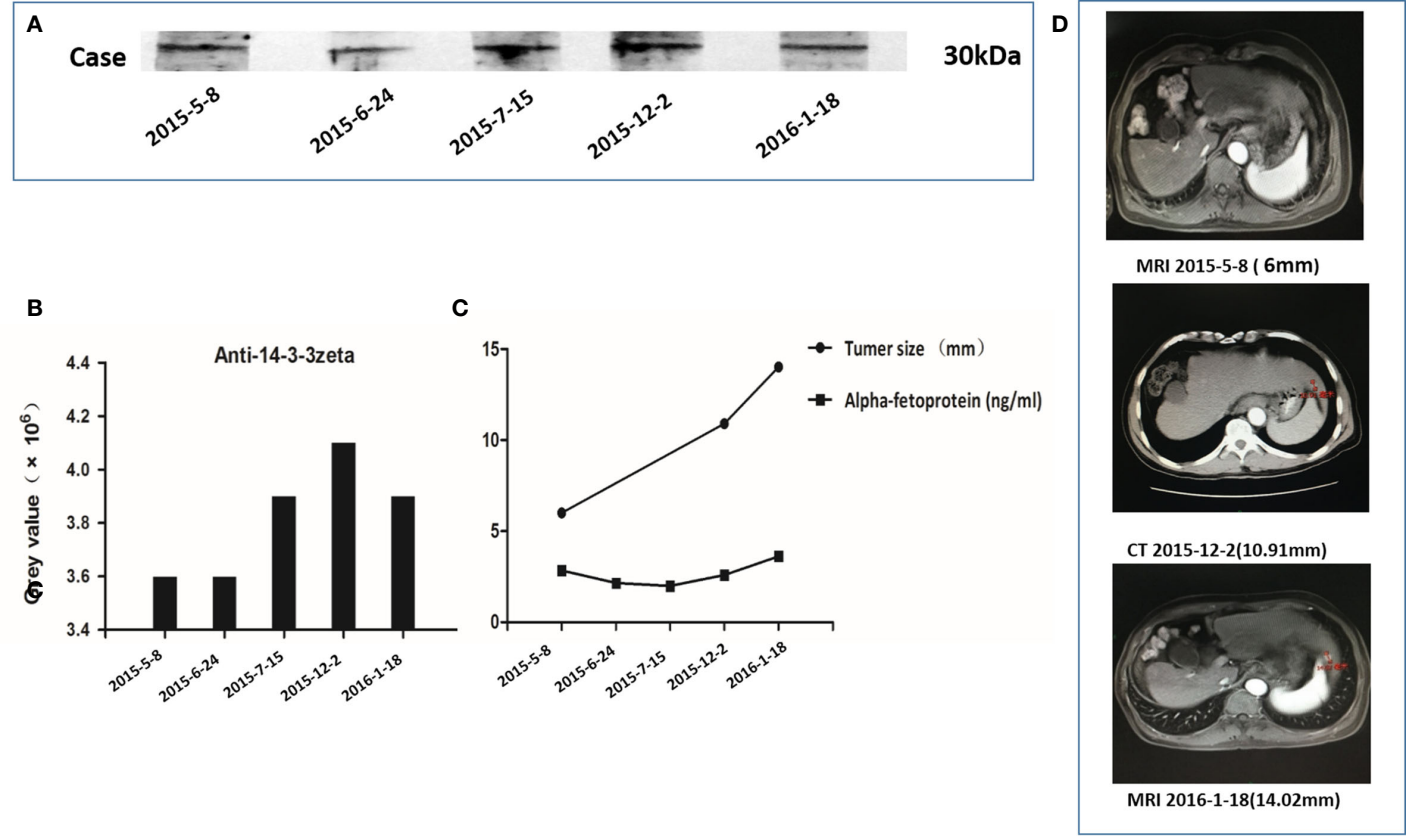
Autoantibody to 14-3-3 zeta in serial serum samples from an alpha-fetoprotein (AFP)-negative hepatocellular carcinoma (HCC) patient. **(A)** The HCC patient was diagnosed on January 18, 2016, with a strong 14-3-3 zeta reactive band. **(B)** During a 9-month period (May 8, 2015, to January 18, 2016), five serum samples obtained from this patient showed a gradual increase in the 14-3-3 zeta reactive band. A stronger reactive band could be observed in the serum 9 months before the diagnosis of HCC. **(C)** The trends in gray values of five serum sample reactive bands were compared with 14-3-3 zeta and AFP values in five serum samples, as well as the tumor nodule size, at three corresponding time points. The gray values and tumor sizes corresponding to the serial serum samples show the same increasing trend. No significant trend in AFP values among the five serum samples was observed, and these values were less than the upper limit of the normal level. **(D)** Imaging data of three corresponding time points of serial serum sampling shows that the size of the nodule gradually increased from 6 to 14.02 mm and was subsequently proven to be a tumor nodule.

Autoantibody to 14-3-3 zeta in serial blood samples from the AFP-negative HCC patient was also tested using Western blotting. The results are shown in **Figure 4**. A 61-year-old AFP-negative HCC patient was diagnosed with HCC on January 18, 2016. In this study, the anti-14-3-3 zeta autoantibody appeared in the serum 9 months before the diagnosis of the tumor and gradually increased as the size of the nodule increased from 6 to 14.02 mm, which was subsequently proven to be a tumor nodule. However, there was no significant trend for AFP values, which were less than the upper limit of the normal level.

### 3.3 Baseline Characteristics of Patients in the LC, CH, and NHS Groups

The baseline characteristics of patients in the LC, CH, and NHS groups are summarized in **Table 1**.

**Table 1:** Baseline characteristics of patients in the LC, CH, and NHS groups.

Variables	LC (*n* = 93)	CH (*n* = 75)	NHS (*n* = 60)
Age (years)	52 ± 13	42 ± 14	39 ± 12
Sex, male/female	70/23	59/16	25/35
HBV/HCV/BC/NBNC	44/23/3/23	56/16/0/3	–
ALT, U/L	37.6 (21.0, 60.7)	76.7 (29.5, 310.4)	–
AST, U/L	48 (31.15, 79.5)	45.6 (25.6, 142.9)	–
TBIL, µmol/L	30.0 (19.95, 51.15)	17.1 (13.1, 38.5)	–
DBIL, µmol/L	7.3 (4.6, 19.75)	4.5 (2.6, 17.2)	–
ALB, g/L	34.2 ± 5.9	41.3 ± 4.7	–
CR, µmol/L	60 (51.3, 70.35)	66.7 (59.5, 73.8)	–
INR	1.2 (1.065, 1.4)	–	–
PT, S	13.5 (11.95, 15.85)	–	–
AFP, ng/ml	4.33 (2.54, 8.935)	–	–
Child–Pugh score	7 (6, 9)	–	–
Child–Pugh grade, A/B/C	61/25/7	–	–
Meld score	11.0 (9, 15)	–	–
Encephalopathy non-/1–2/3–4	85/7/1	–	–
Ascites degree none/low/medium/high	34/49/7/3	–	–

Continuous variables are expressed as the mean ± SD or medians (25th and 75th percentiles); count data are described as frequency.

LC, liver cirrhosis; CH, chronic hepatitis; NHS, normal human sera.

### 3.4 Frequency and Titer of Autoantibody Against 14-3-3 Zeta in Patients With LC

**Figure 5:**
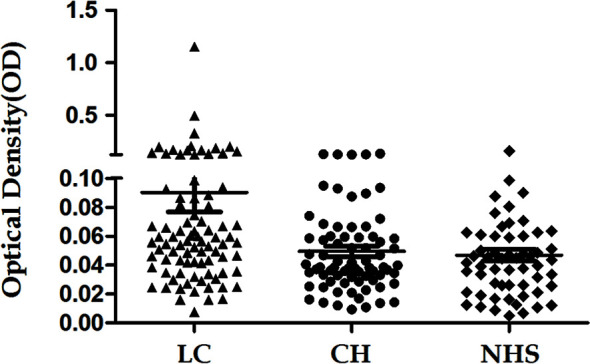
Titer of autoantibody against 14-3-3 zeta in human sera determined by ELISA. The range of antibody titers to 14-3-3 zeta was expressed as that obtained by ELISA. The mean ± 3 SD of normal human serum (NHS) samples is shown in relation to values for all serum samples. The titer of anti-14-3-3 zeta autoantibody in liver cirrhosis (LC) was much higher than that in other types of sera (*p* = 0.000).

**Figure 6:**
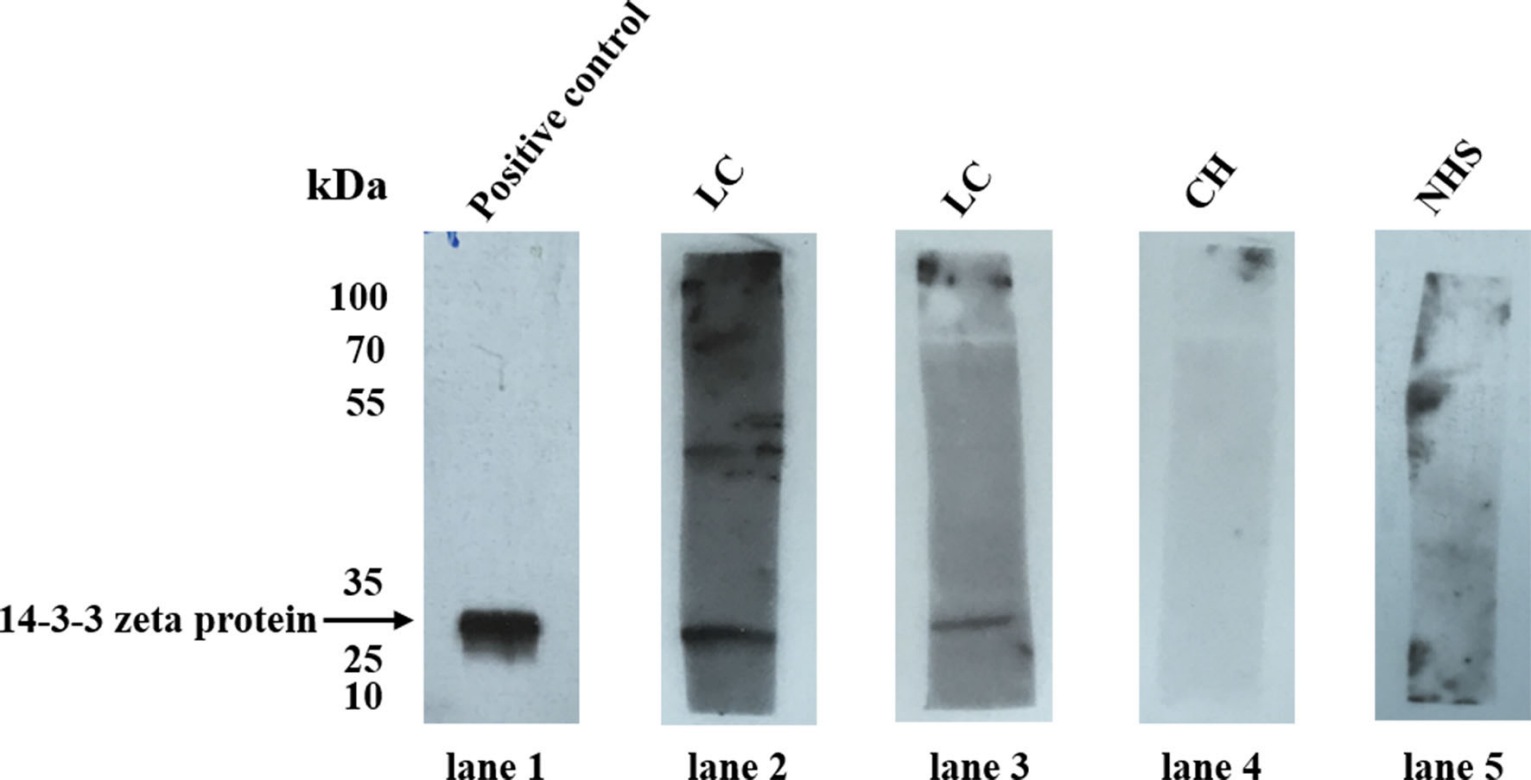
Western blot analysis of representative sera assessed by ELISA. Lane 1, the polyclonal anti-14-3-3 zeta autoantibody was used as a positive control; lanes 2 and 3, two representative liver cirrhosis (LC) serum samples that were positive by ELISA also showed strong reactivity with 14-3-3 zeta recombinant protein by Western blot analysis; lanes 4 and 5, randomly selected chronic hepatitis (CH) sera and normal human serum (NHS) samples, respectively, with negative reactivity to 14-3-3 zeta recombinant protein.

The 14-3-3 zeta recombinant protein was used as a coating antigen in enzyme-linked immunosorbent assay (ELISA) to screen for an autoantibody against 14-3-3 zeta in sera from patients with LC, CH, and NHS. As shown in **Table 2**, the prevalence of autoantibodies against 14-3-3 zeta was 16.1% (15/93) in LC, which was significantly higher than that in CH and NHS (LC *versus* CH, *p* = 0.000; LC and NHS, *p* = 0.01). The titers of anti-14-3-3 zeta autoantibody in sera from the three groups are shown in **Figure 5**. The average titer of anti-14-3-3 zeta autoantibody in sera from LC patients was much higher than that in CH patients and NHS (LC *versus* CH, *p* = 0.000; LC and NHS, *p* = 0.000). These results were confirmed using Western blot analysis. **Figure 6** shows that representative LC sera with a positive reaction to 14-3-3 zeta based on ELISA showed stronger reactivity in Western blotting than CH and normal sera.

**Table 2:** Frequency of autoantibody against 14-3-3 zeta in human sera based on ELISA.

Type of sera	No. of tested	Autoantibody to 14-3-3 zeta (%)	*p*-value
LC	93	15 (16.1)	–
CH	75	0 (0)	0.001 (LC *versus* CH)
NHS	60	1 (1.7)	0.01 (LC *versus* NHS)

Cutoff value, mean + 3 SD of NHS.

LC, liver cirrhosis; CH, chronic hepatitis; NHS, normal human sera.

### 3.5 Detection of Intense Nuclear Staining Pattern in Hep2 Cells Using Indirect Immunofluorescence Assay With Representative Positive LC Sera

**Figure 7:**
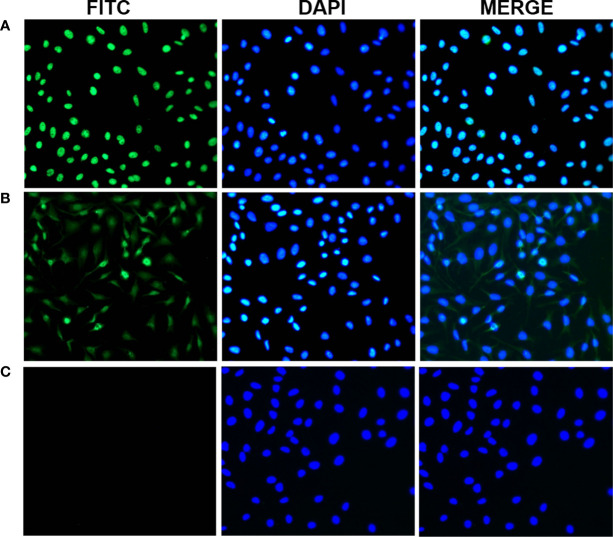
Representative immunofluorescence staining pattern of anti-14-3-3 zeta autoantibody with positive liver cirrhosis (LC) serum. **(A)** A polyclonal anti-14-3-3 zeta antibody that showed a cytoplasmic immunofluorescence staining pattern was used as a positive control. **(B)** A representative anti-14-3-3 zeta autoantibody-positive LC serum sample demonstrated an intense staining pattern. **(C)** normal human serum (NHS) was used as a negative control.

To further confirm the reactivity of the 14-3-3 zeta autoantibody in LC sera and the intracellular location of 14-3-3 zeta, commercially purchased Hep2 cell slides were used in an indirect immunofluorescence assay to detect LC sera with a positive anti-14-3-3 zeta autoantibody, as shown by ELISA. As shown in **Figure 7**, representative anti-14-3-3 zeta autoantibody-positive LC sera resulted in an intense cytoplasmic staining pattern, which was similar to the fluorescent staining pattern and cellular location shown with the polyclonal anti-14-3-3 zeta autoantibody.

## 4 Discussion

Many studies have identified the presence of autoantibodies in human sera, not only in systemic autoimmune diseases such as systemic lupus erythematosus (SLE) and rheumatoid arthritis (RA) (14–16), but also in non-autoimmune diseases such as cancer (17, 18). Although the mechanism underlying the production of such autoantibodies remains poorly understood, they are commonly used as invaluable tools for clinical detection in some diseases (19–22). In recent years, many novel TAAs and autoantibodies, such as p53, p62, and p90, have been isolated and characterized from patients with HCC (23–26). These are potential biomarkers for the early diagnosis of HCC, and a mini-array of multiple TAAs can enhance autoantibody detection for the diagnosis of HCC (27, 28). In addition, studies have shown that some autoantibodies to TAAs can be observed in the serum 6–9 months before the clinical diagnosis of HCC (29, 30), which means that autoantibodies to TAAs might predict hepatocarcinogenesis. Currently, more than 60% of HCC patients cannot obtain timely diagnosis and treatment, which leads to a high mortality rate, especially for very early HCC and AFP-negative HCC. Therefore, screening novel autoantibodies to TAAs for AFP-negative HCC and very early HCC is still an important task.

In this study, we aimed to screen and identify novel proteins in AFP-negative HCC and evaluated the feasibility of using the autoantibodies to those proteins to predict hepatocarcinogenesis. Firstly, we screened and identified DEPs between AFP-negative HCC tissue and adjacent non-tumor liver tissue using SWATH-MS proteome technology, which included 14-3-3 zeta protein, a protein-binding protein. The 14-3-3 proteins are a group of highly conserved acidic proteins encoded by different genes. There are seven distinct isoforms, β, γ, ϵ, ζ, η, σ, and τ, in mammals. The 14-3-3 isoforms have been linked to carcinogenesis, as they can be found in conjunction with various target proteins *via* phosphorylation-dependent or non-dependent phosphorylation reactions, and are involved in all human physiological reactions, including cell cycle regulation, signal transduction, apoptosis, proliferation, and differentiation (31). Studies have shown that 14-3-3 zeta, a 14-3-3 protein family member, is overexpressed in various tumor types, including HCC tissue samples (32, 33). In one study, the researchers investigated the association between serum 14-3-3 isoforms and HCC progression and prognosis. They found that serum 14-3-3 β is a potential biomarker for the diagnosis of early-stage HCC and associated with metastasis and poor prognosis. There was no statistical difference in the serum levels of 14-3-3 ϵ, γ, and ζ between HCC and control groups (34). However, our previous studies have shown that the level of anti-14-3-3 zeta autoantibody is significantly higher in the sera of patients with HCC than in those of patients with other chronic liver diseases and NHS. In addition, anti-14-3-3 zeta autoantibody was detected 9 months before the clinical diagnosis of HCC in several patients *via* serial blood sampling (35). These results suggest that anti-14-3-3 zeta autoantibody is a potential biomarker for early-stage HCC screening and diagnosis. However, there are few screening studies on AFP-negative HCC and very early HCC.

By further serological verification, one patient was diagnosed with AFP-negative HCC. The outcome of serial serum sampling showed that the anti-14-3-3 zeta autoantibody appeared in sera 9 months before the diagnosis of HCC and gradually increased as the size of the nodule increased, which was subsequently proven to be a tumor nodule. However, there was no significant trend in AFP values. The levels of AFP in five serial serum samples were less than the upper limit of the normal level. The results suggest that AFP might not be an ideal biomarker for the detection of HCC. Several studies have shown that the sensitivity and specificity of AFP for HCC diagnosis is not optimal, as 40% of HCC cases were not detected by screening for AFP (36, 37). The findings in the present study suggest that positive anti-14-3-3 zeta autoantibody might be a biomarker of carcinogenesis in AFP-negative HCC patients. This confirms our previous findings that indicate that anti-14-3-3 zeta autoantibody could be a potential biomarker for early HCC diagnosis. Concerning AFP-negative HCC patients, the presence of an anti-14-3-3 zeta autoantibody can be detected far earlier than the manifestation of disease based on imaging.

Studies on the level of serum autoantibodies during liver fibrogenesis have shown that this is a potential method for the identification of biomarkers in premalignant liver disease. For example, metallopeptidase inhibitor 1 (TIMP-1), α-2-macroglobulin, and hyaluronic acid (HA) have been identified as serum molecules (38, 39). However, these markers are not liver-specific. To date, few studies have been performed using autoantibodies as biomarkers in premalignant liver disease to identify those who might be at risk of developing HCC. The appearance of anti-14-3-3 zeta autoantibody in the LC stage in patients with HCC indicates that anti-14-3-3 zeta autoantibody might be predictive of hepatocarcinogenesis in premalignant liver disease. Based on these findings, we attempted to detect the prevalence of serum autoantibodies against 14-3-3 zeta in premalignant liver disease. We found that the prevalence of autoantibodies against 14-3-3 zeta protein was significantly higher than that in chronic hepatitis and normal human sera. Therefore, we suggest that anti-14-3-3 zeta autoantibody could be a biomarker to predict hepatocarcinogenesis. Further follow-up of and research on patients with positive autoantibodies will be continued to confirm the relationship between anti-14-3-3 zeta autoantibody and hepatocarcinogenesis.

One limitation of this study is that the 14-3-3 zeta construct, GST-14-3-3 WT (plasmid ID: 1944), in this study was created from mouse origin because a human origin 14-3-3 zeta plasmid could not be obtained. Actually, this should not influence the results because human 14-3-3 zeta and mouse 14-3-3 zeta are highly homologous with only one amino acid difference. In addition, more time is needed to follow up on the outcome of patients with positive autoantibodies, and the serological changes in these patients should be observed to further determine the correlation with carcinogenesis.

## Conclusion

In conclusion, the comprehensive use of serum biomarkers is a promising method to predict the development of HCC, especially early and AFP-negative liver cancer. Our study found that serum anti-14-3-3 zeta autoantibody is a potential non-invasive serum biomarker for predicting hepatocarcinogenesis, which might significantly improve the diagnosis efficiency for early and AFP-negative liver cancer and reduce the associated fatality rate to a certain extent.

## Data Availability Statement

The original contributions presented in this study are included in the article/**Supplementary Material**. Further inquiries can be directed to the corresponding author. The mass spectrometry proteomics data have been deposited to the ProteomeXchange Consortium via the PRIDE partner repository with the dataset identifier PXD028854.

## Ethics Statement

This study was approved by the Institutional Review Board of Capital Medical University, Beijing, China. The patients/participants provided their written informed consent to participate in this study.

## Author Contributions

All authors listed have made a substantial, direct, and intellectual contributions to the work and approved it for publication. All authors contributed to the article and approved the submitted version.

## Funding

This work was supported by the Beijing Municipal Administration of Hospitals Clinical Medicine Development of Special Funding Support (XMLX201830, ZYLX202125), Grant of High Technical Personnel Training Item from Beijing Health System (2015–3–104), Scientific Research Common Program of Beijing Municipal Commission of Education (KM201610025021), and the National Natural Science Foundation of China (81770611).

## Conflict of Interest

The authors declare that the research was conducted in the absence of any commercial or financial relationships that could be construed as a potential conflict of interest.

## Publisher’s Note

All claims expressed in this article are solely those of the authors and do not necessarily represent those of their affiliated organizations, or those of the publisher, the editors and the reviewers. Any product that may be evaluated in this article, or claim that may be made by its manufacturer, is not guaranteed or endorsed by the publisher.

## Supplementary Material

The Supplementary Material for this article can be found online at: https://www.frontiersin.org/articles/10.3389/fonc.2021.733680/full#supplementary-material
