# Supplementary figures and images for: Serum Anti-14-3-3 Zeta Autoantibody as a Biomarker for Predicting Hepatocarcinogenesis
Source: Front Oncol. 2021 Oct 15;11:733680. doi: 10.3389/fonc.2021.733680 (PMC8555665; doi:10.3389/fonc.2021.733680)

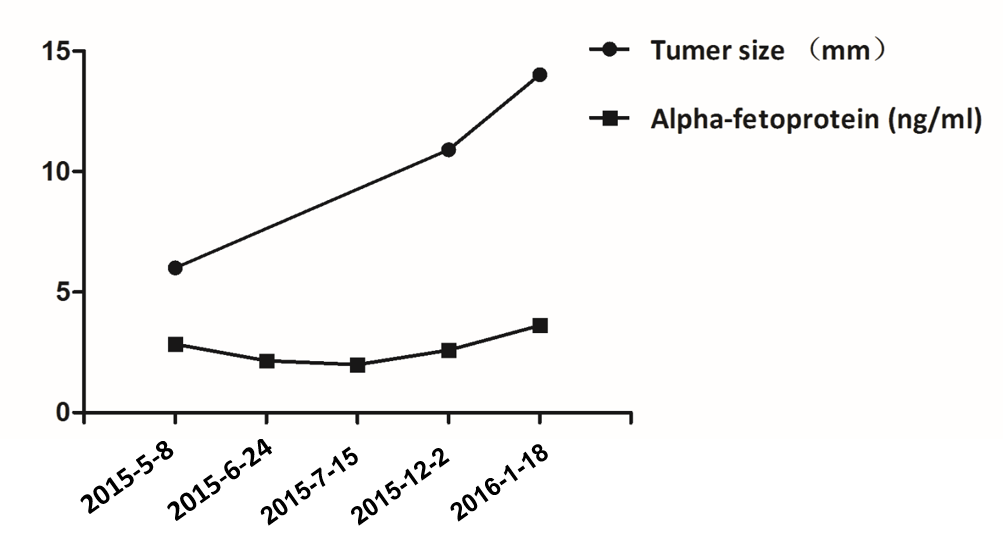

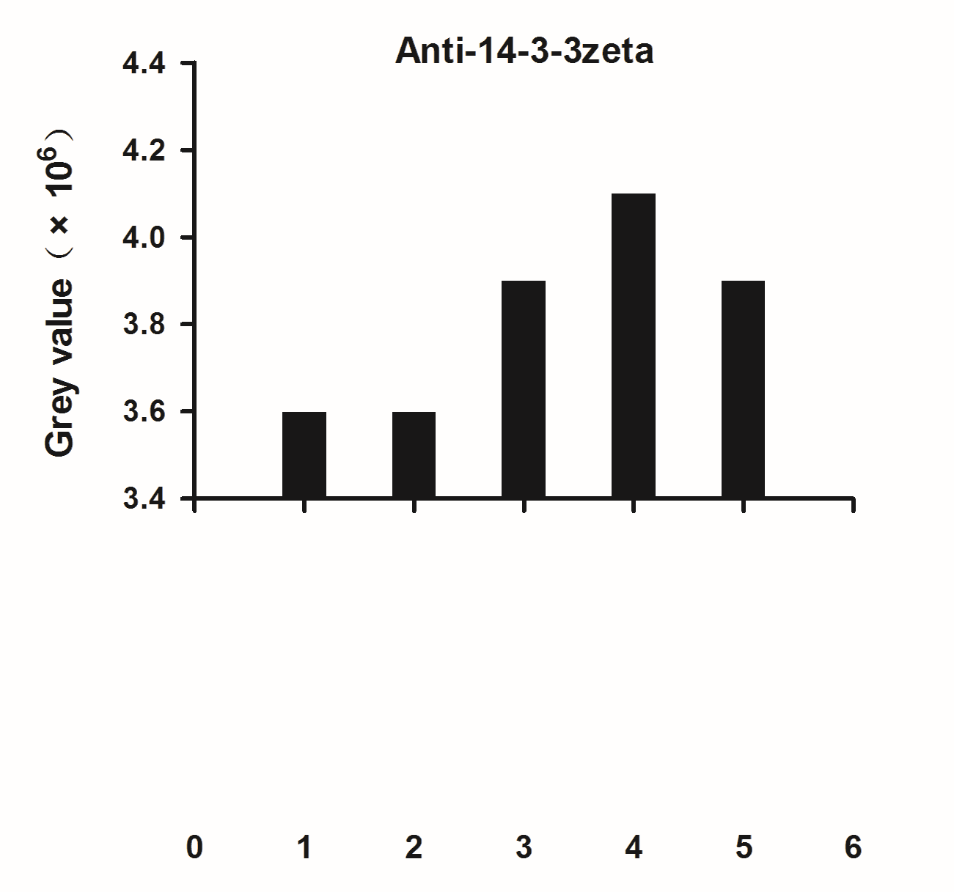

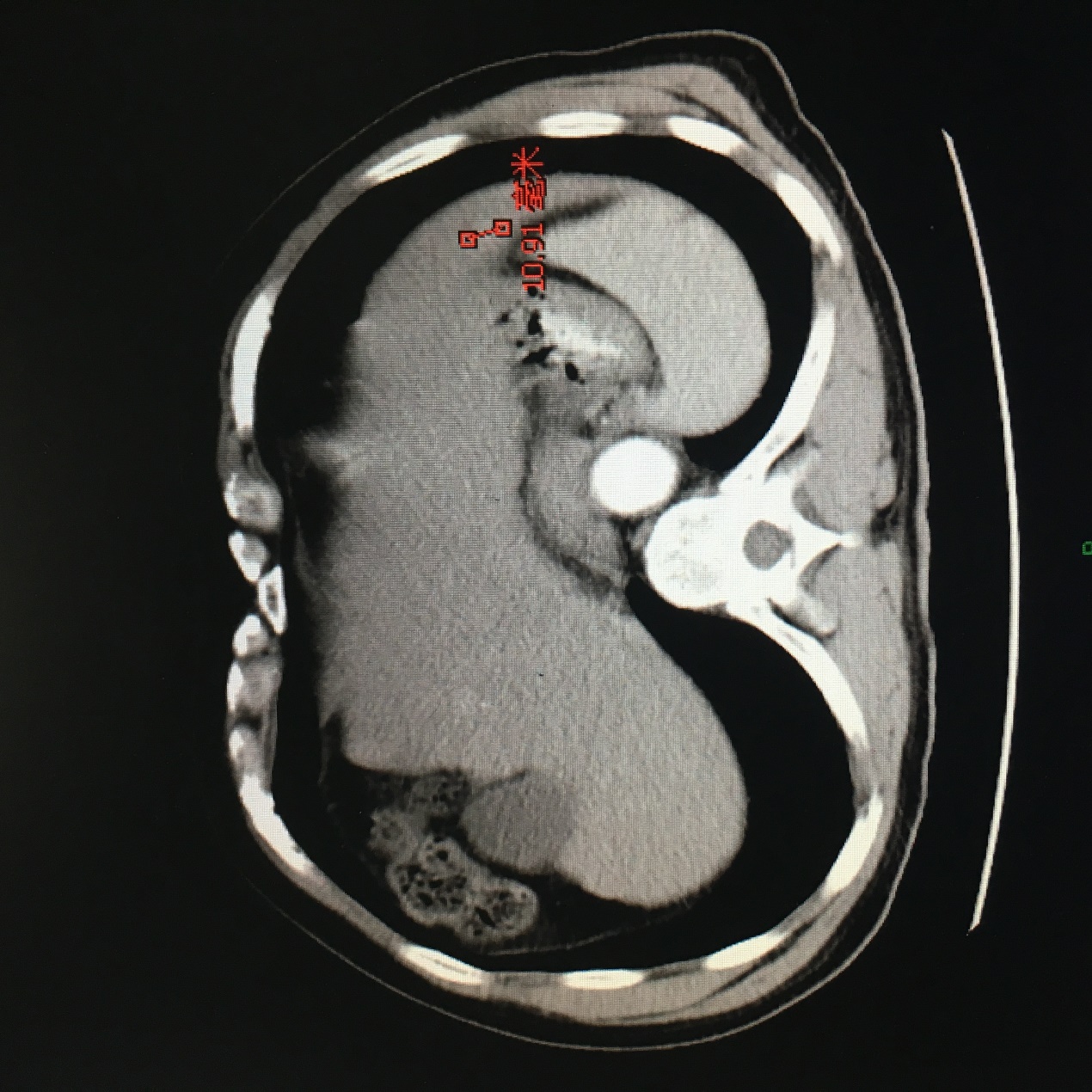

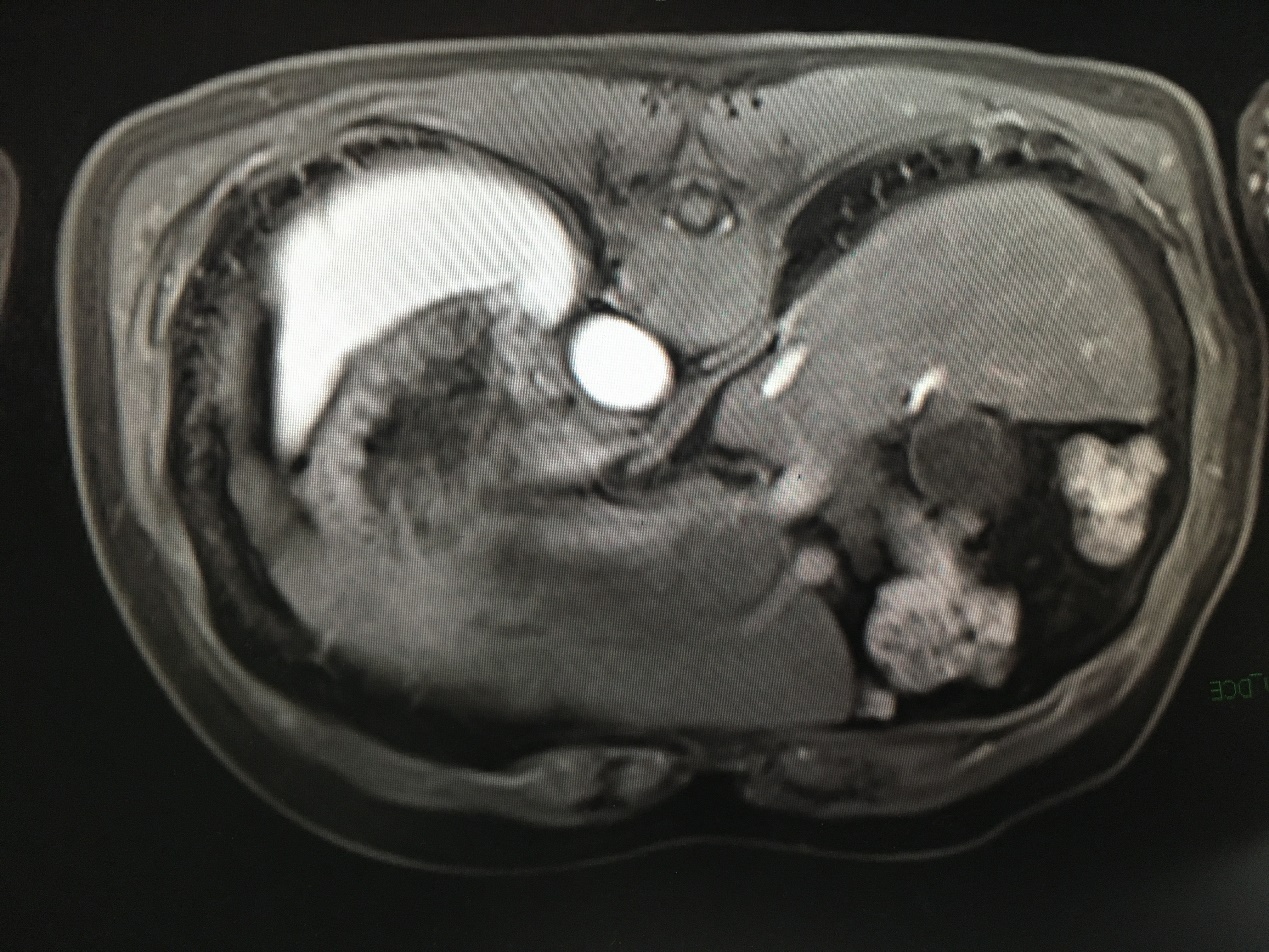

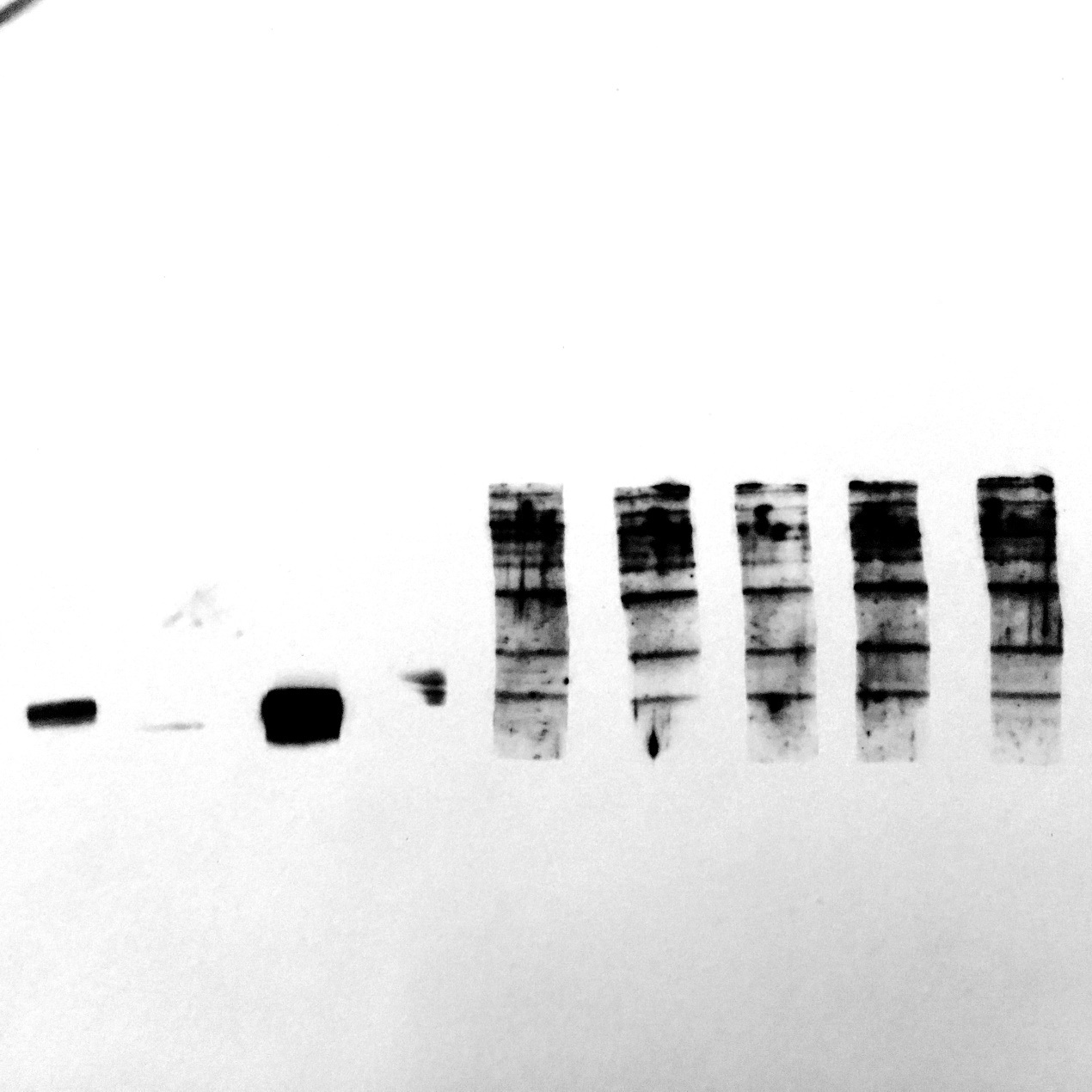


14-3-3zeta protein autobody for WB


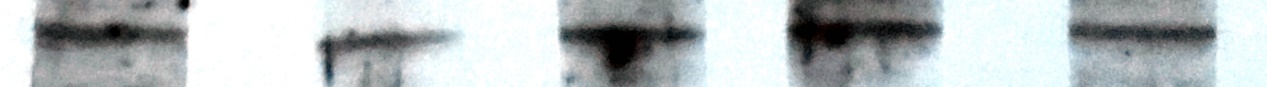

Supplement: Supplementary file 1 [file DataSheet_1.zip › Raw data/raw data for figure 4/raw data of serum series.docx]

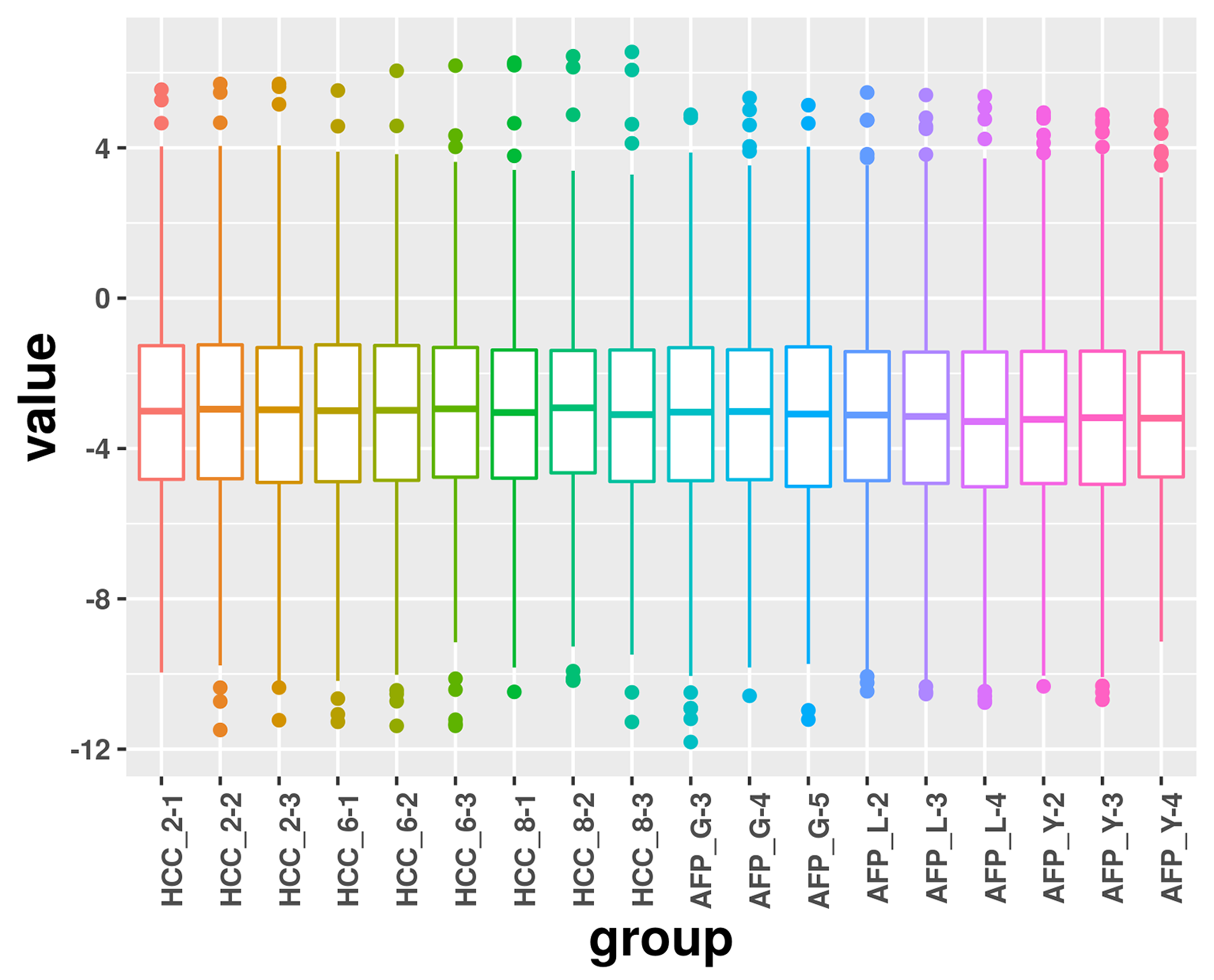

Supplement: Supplementary file 2 [file DataSheet_2.zip › Raw figure/raw image of Figure 1/FIGURE 1A.tif]

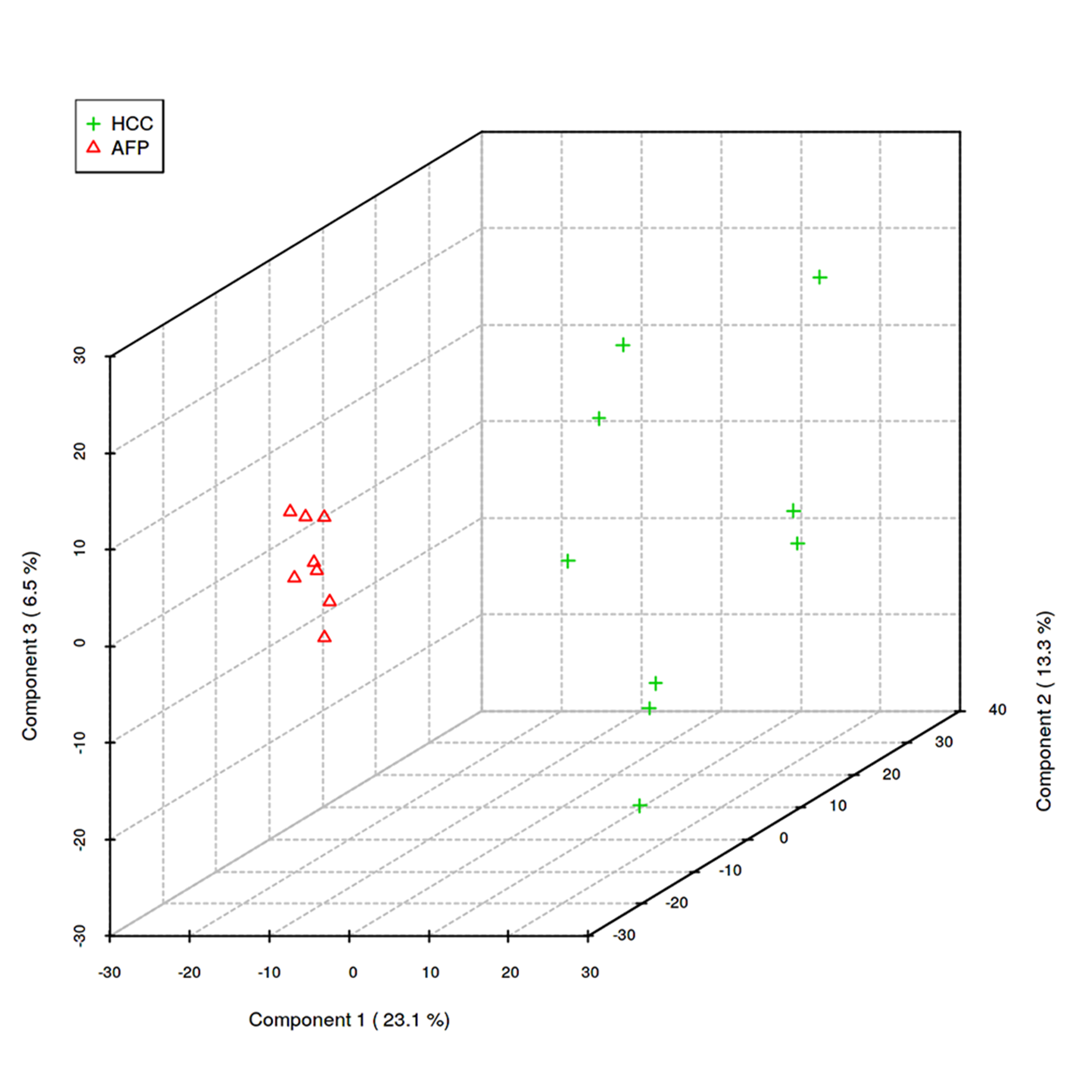

Supplement: Supplementary file 2 [file DataSheet_2.zip › Raw figure/raw image of Figure 1/FIGURE 1B.tif]

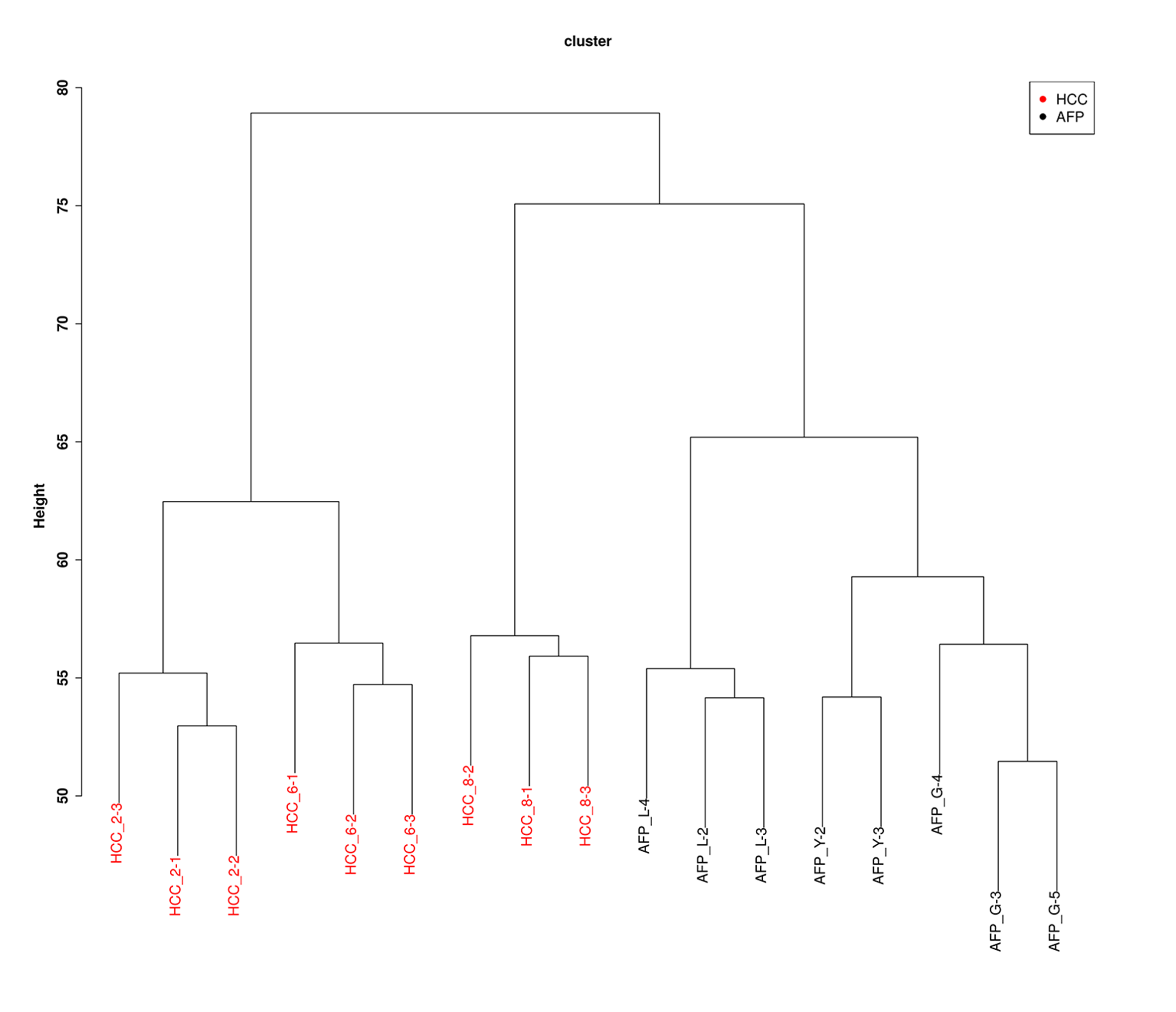

Supplement: Supplementary file 2 [file DataSheet_2.zip › Raw figure/raw image of Figure 1/FIGURE 1C.tif]

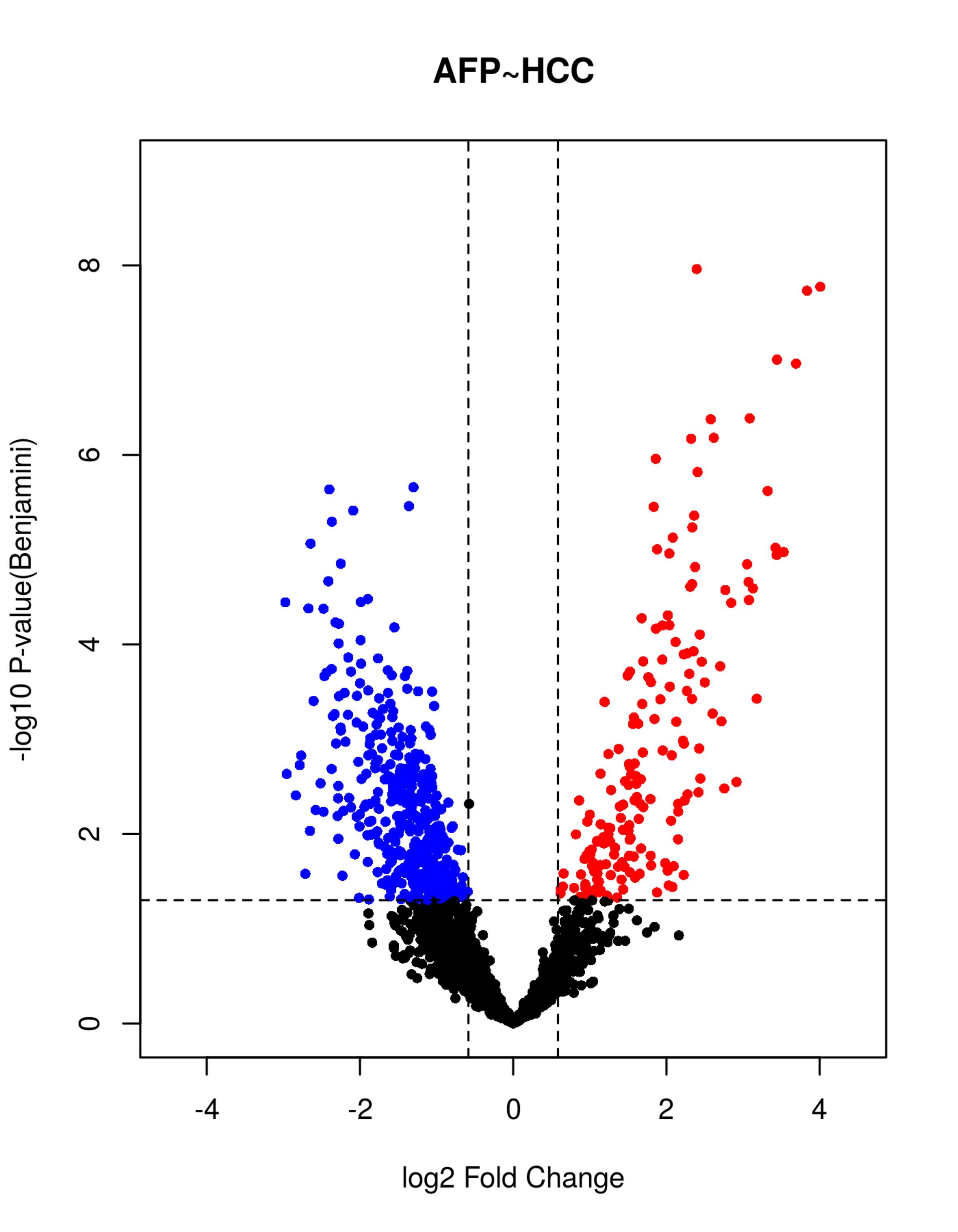

Supplement: Supplementary file 2 [file DataSheet_2.zip › Raw figure/raw image of Figure 1/FIGURE 1D.tif]

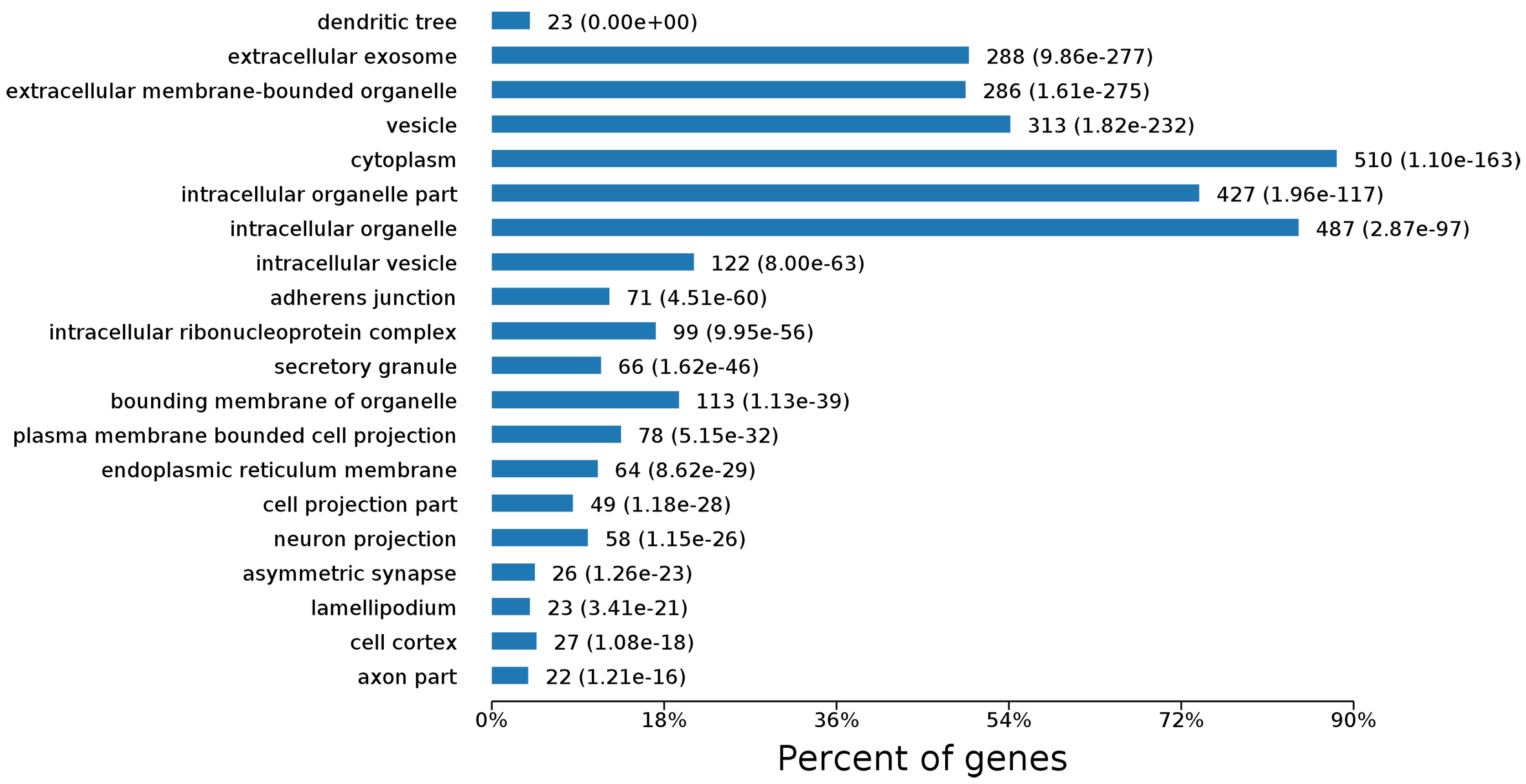

Supplement: Supplementary file 2 [file DataSheet_2.zip › Raw figure/raw image of Figure 2/FIGURE 2A.tif]

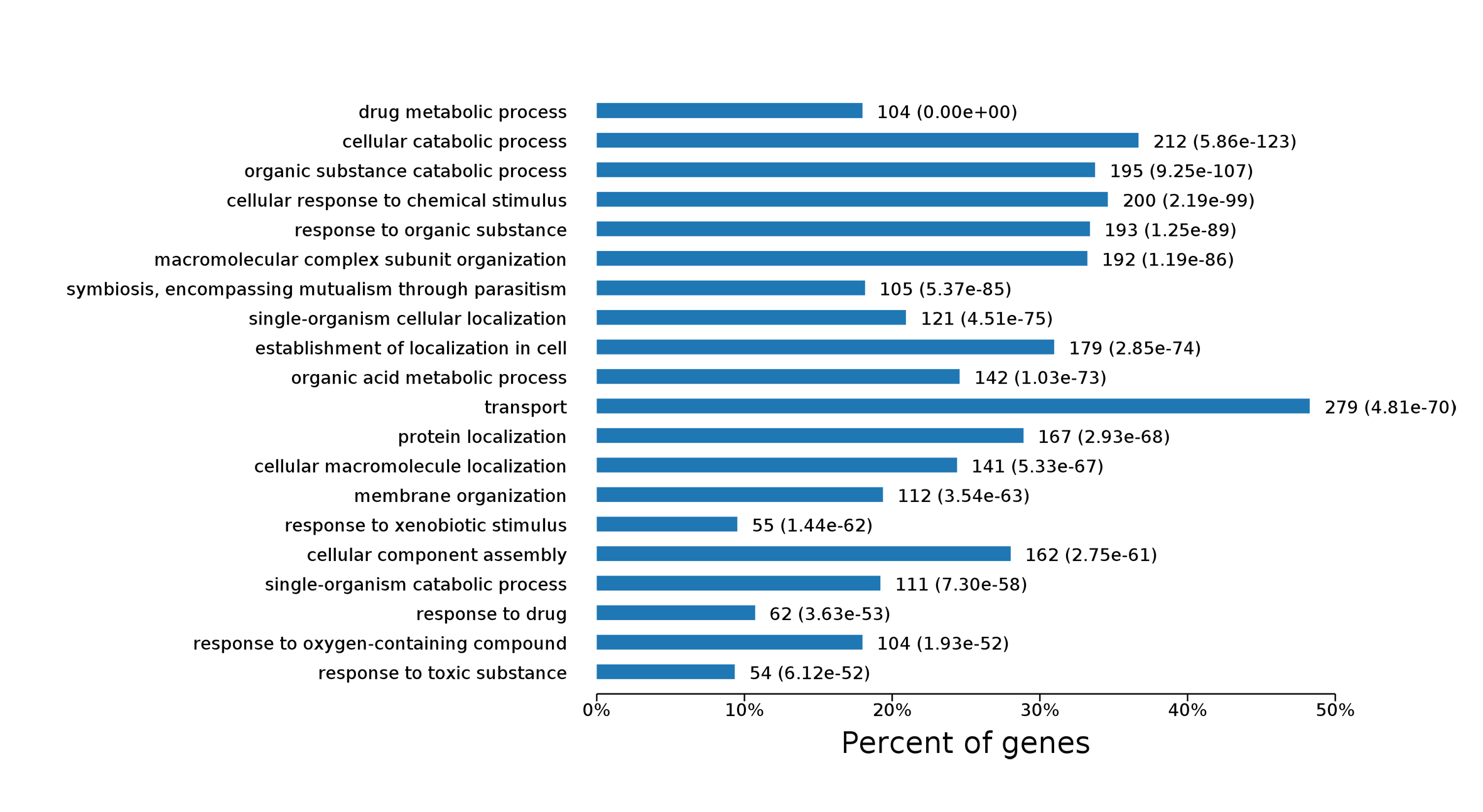

Supplement: Supplementary file 2 [file DataSheet_2.zip › Raw figure/raw image of Figure 2/FIGURE 2B.tif]

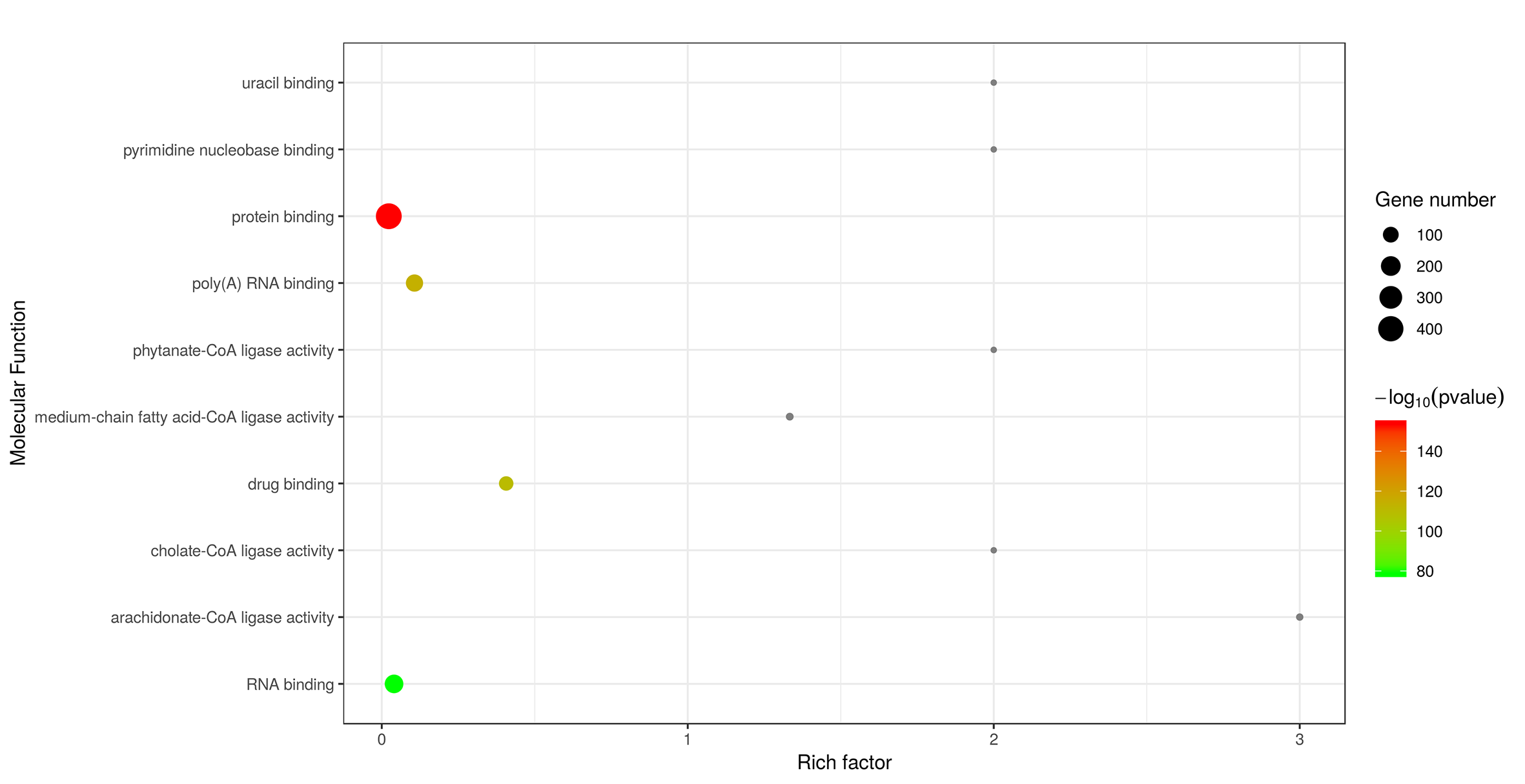

Supplement: Supplementary file 2 [file DataSheet_2.zip › Raw figure/raw image of Figure 2/FIGURE 2C.tif]

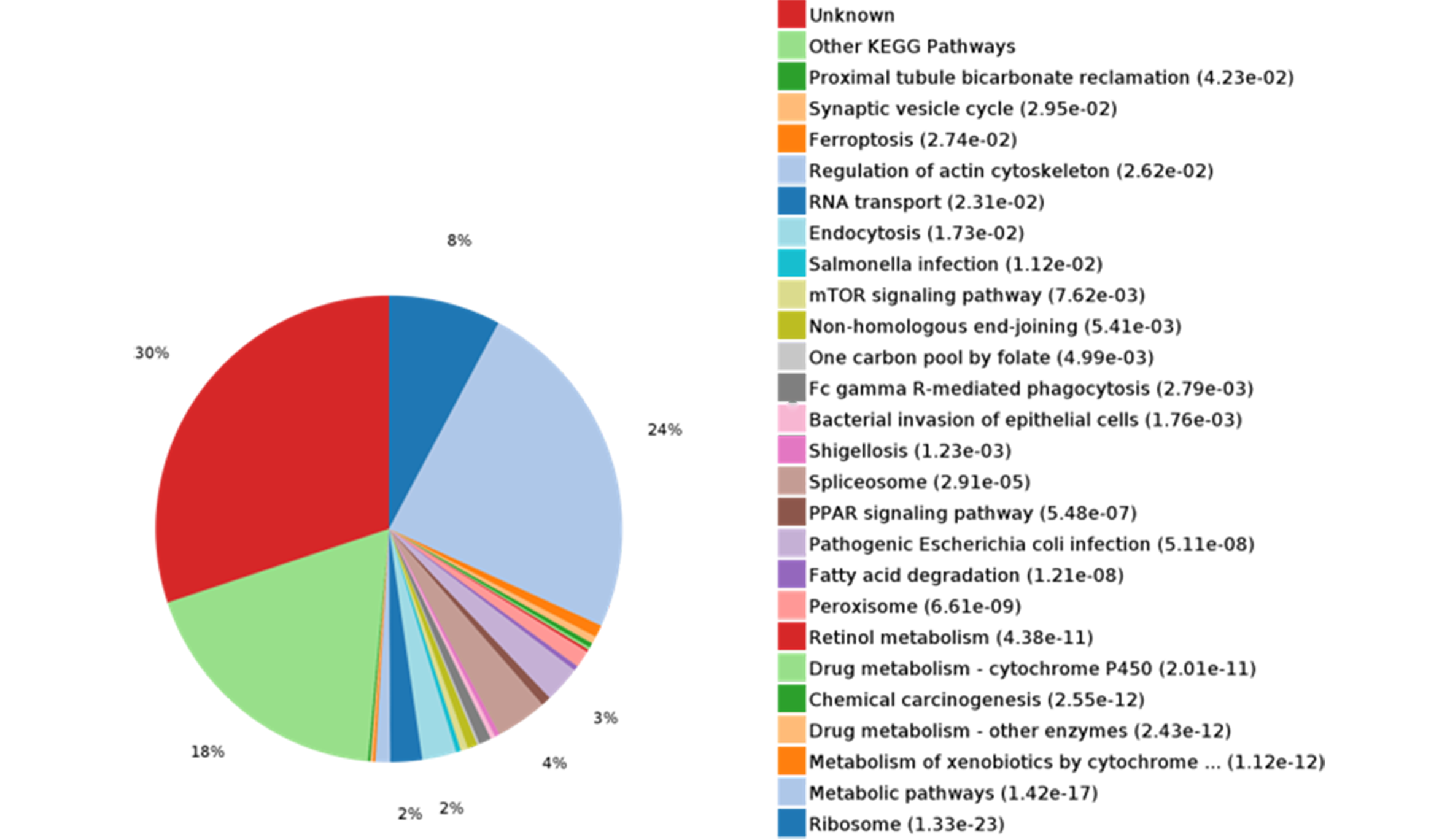

Supplement: Supplementary file 2 [file DataSheet_2.zip › Raw figure/raw image of Figure 3/FIGURE 3.tif]

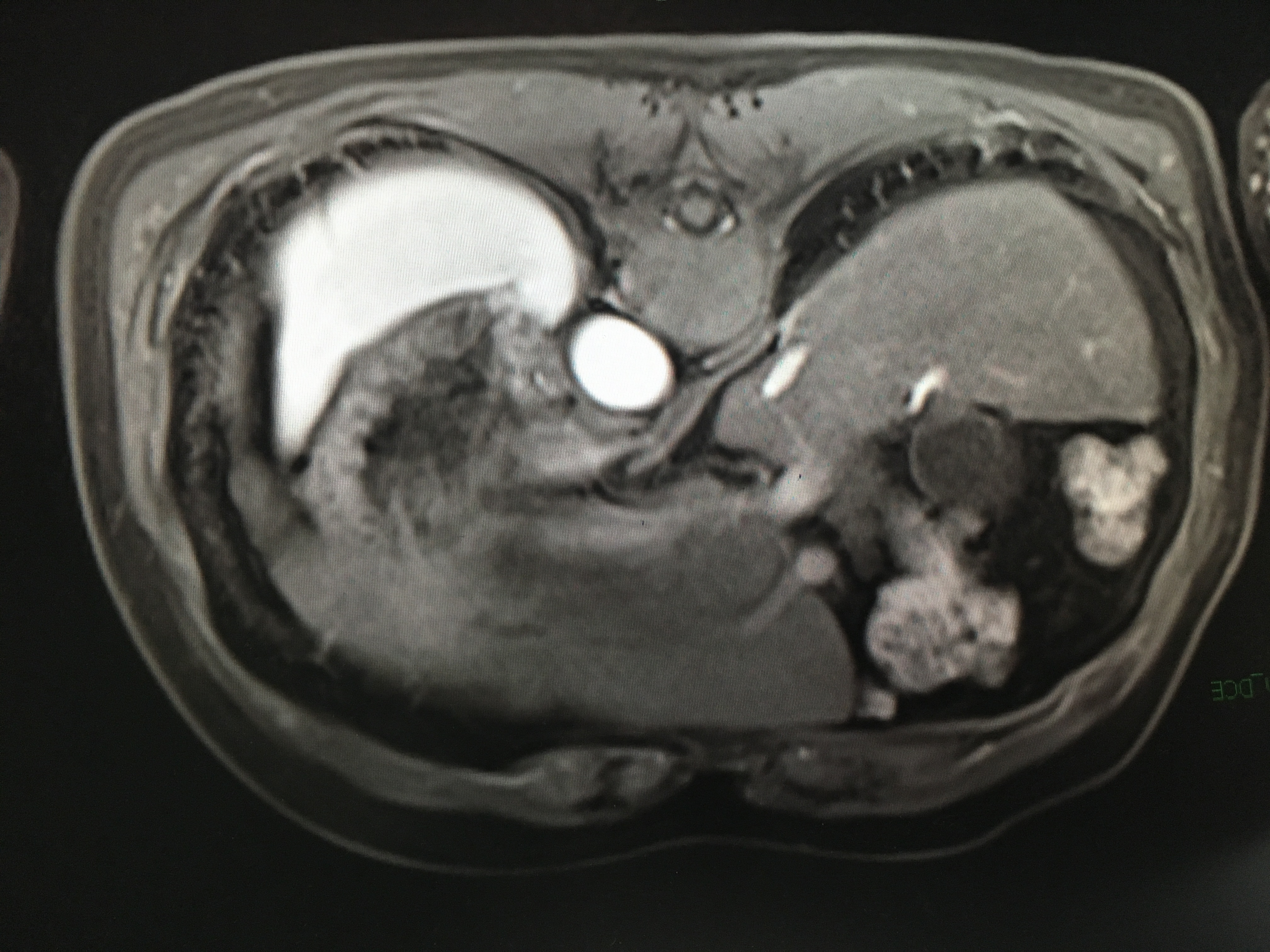

Supplement: Supplementary file 2 [file DataSheet_2.zip › Raw figure/raw image of Figure 4/2015-05-08 MRI.JPG]

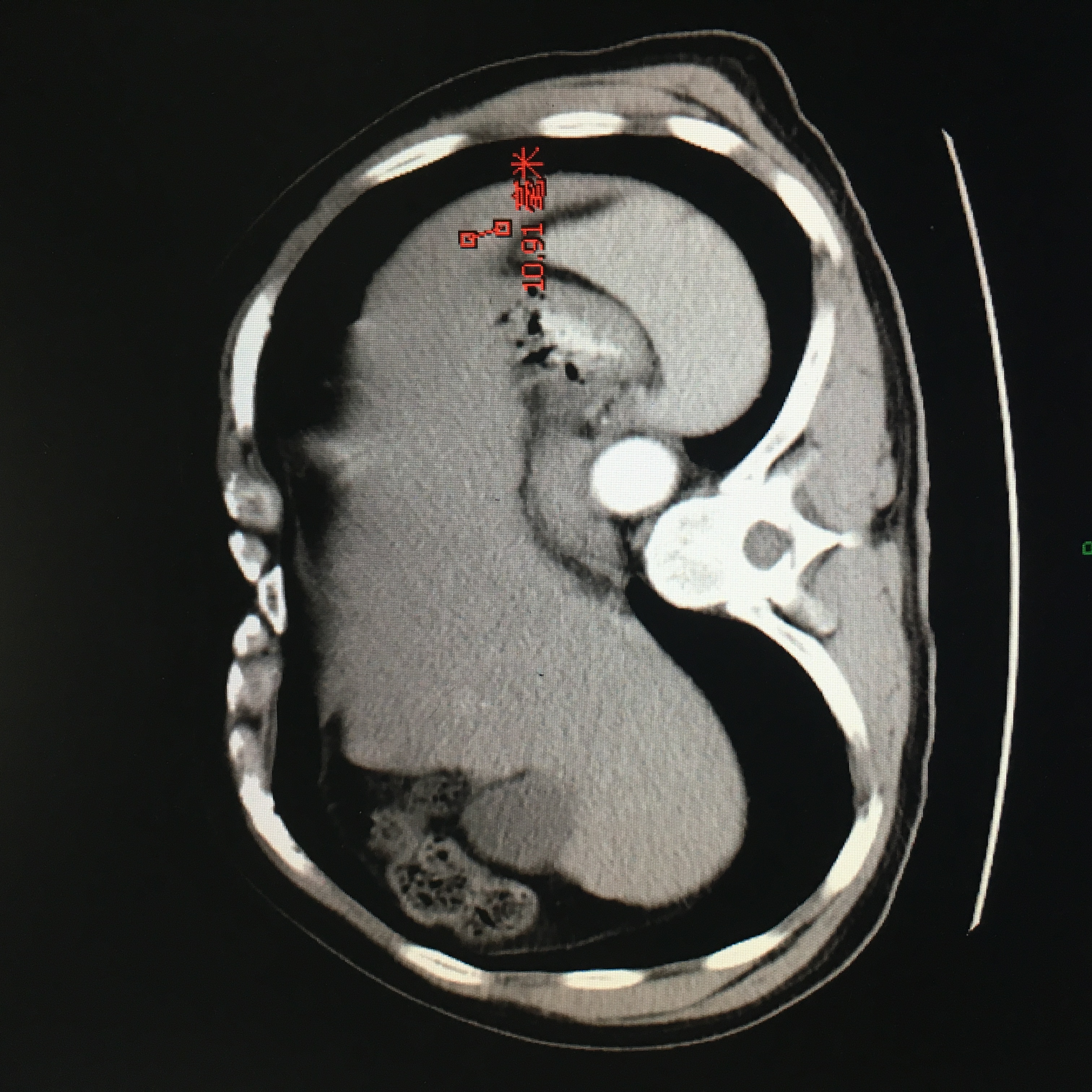

Supplement: Supplementary file 2 [file DataSheet_2.zip › Raw figure/raw image of Figure 4/2015-12-2 CT.JPG]

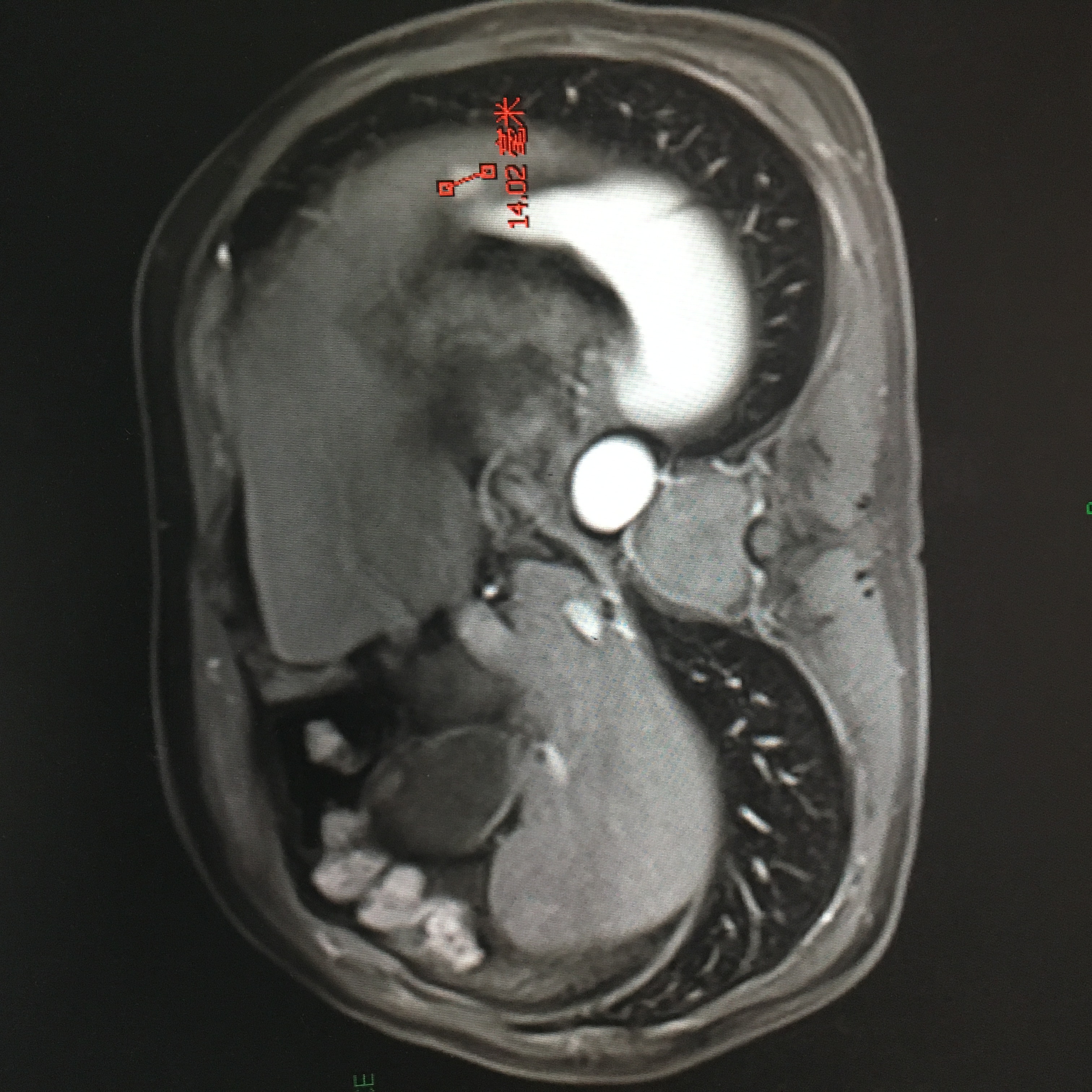

Supplement: Supplementary file 2 [file DataSheet_2.zip › Raw figure/raw image of Figure 4/2016-01-18 MRI.JPG]

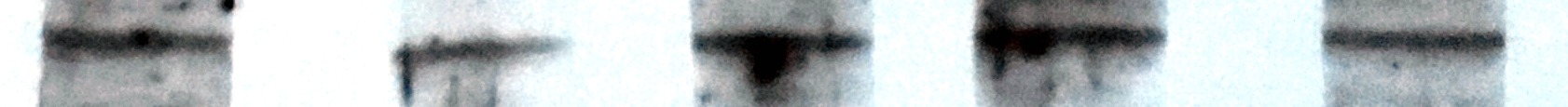

Supplement: Supplementary file 2 [file DataSheet_2.zip › Raw figure/raw image of Figure 4/WB of serial serum (2).jpg]

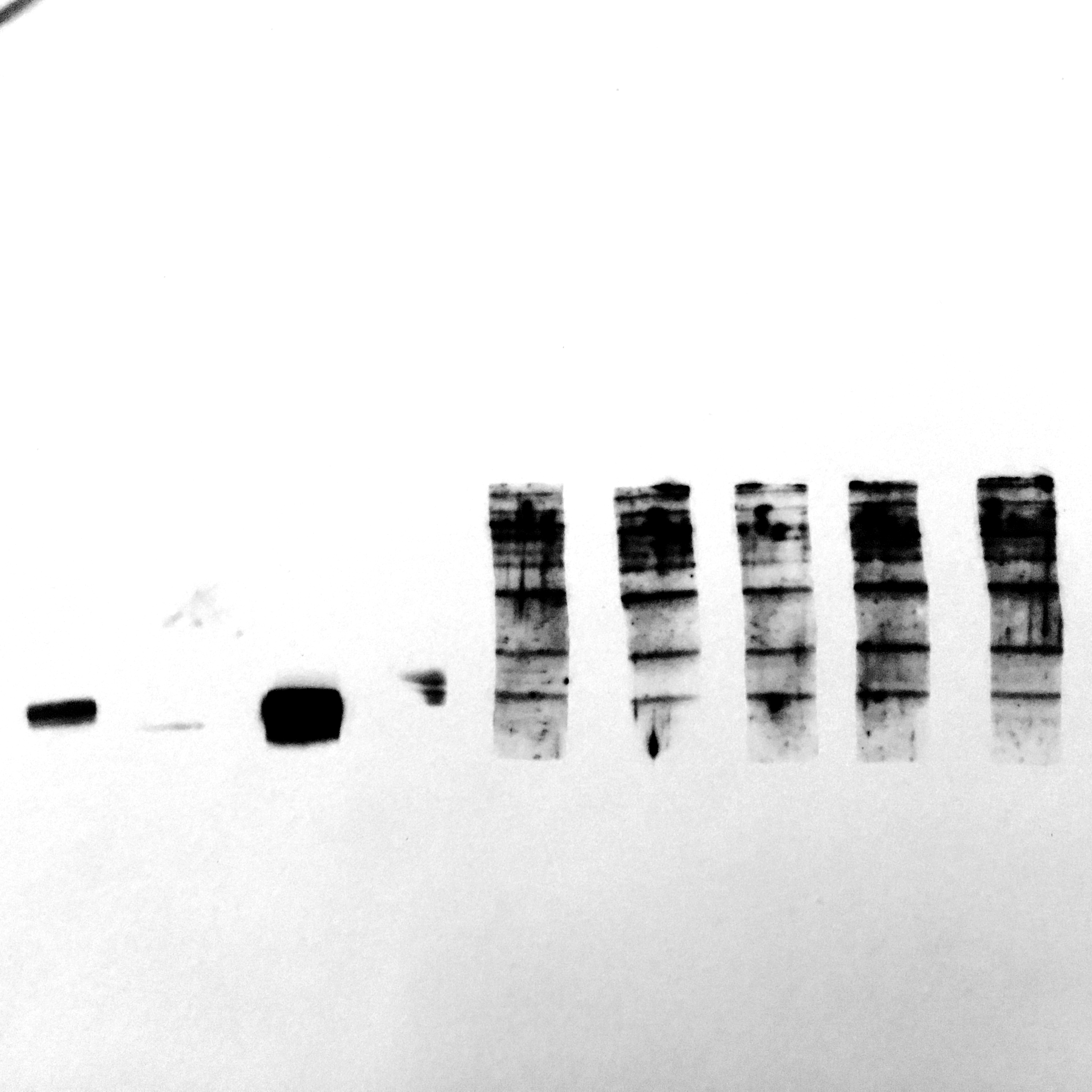

Supplement: Supplementary file 2 [file DataSheet_2.zip › Raw figure/raw image of Figure 4/WB of serial serum.jpg]

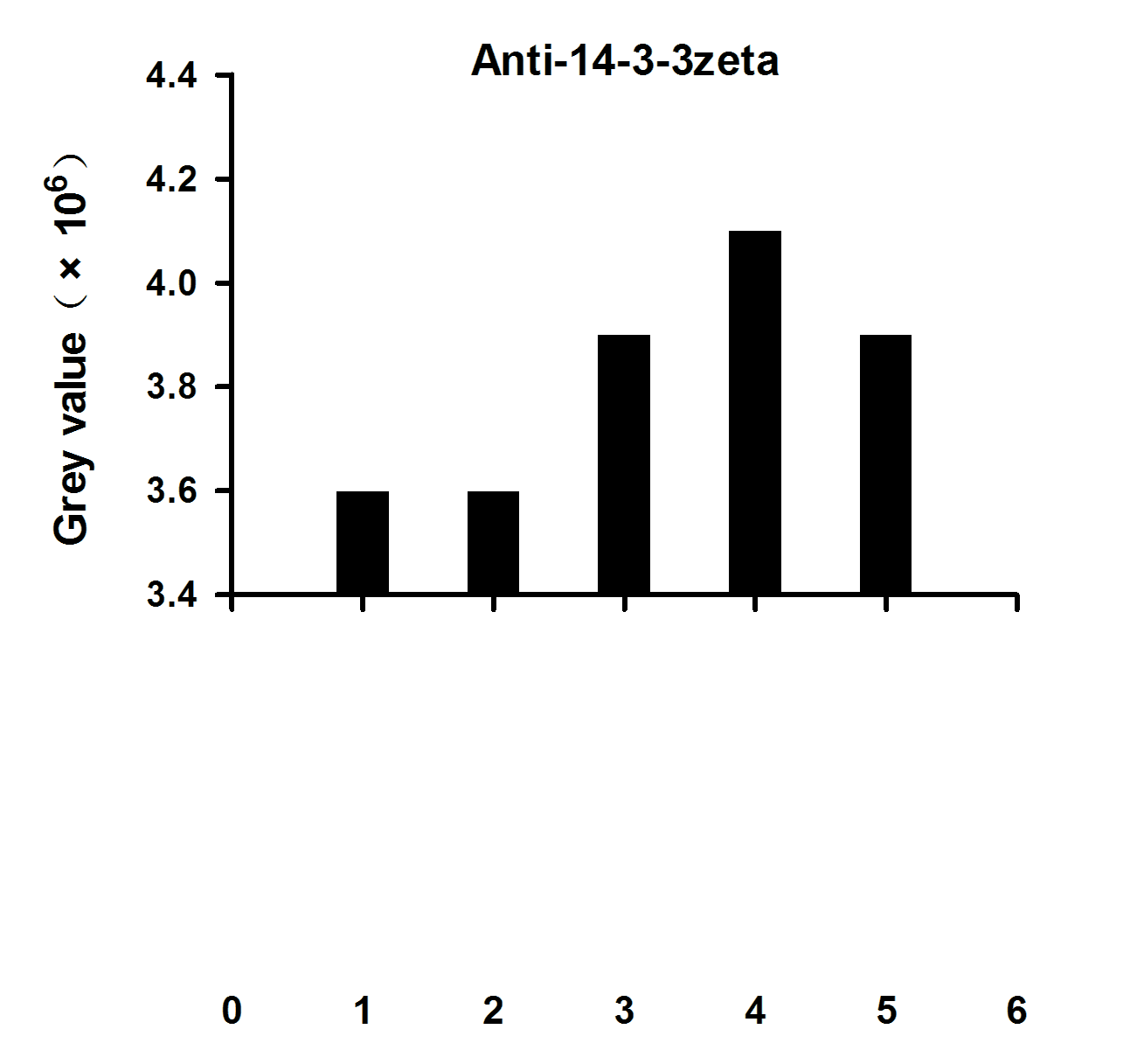

Supplement: Supplementary file 2 [file DataSheet_2.zip › Raw figure/raw image of Figure 4/level of 1433 Ab.tif]

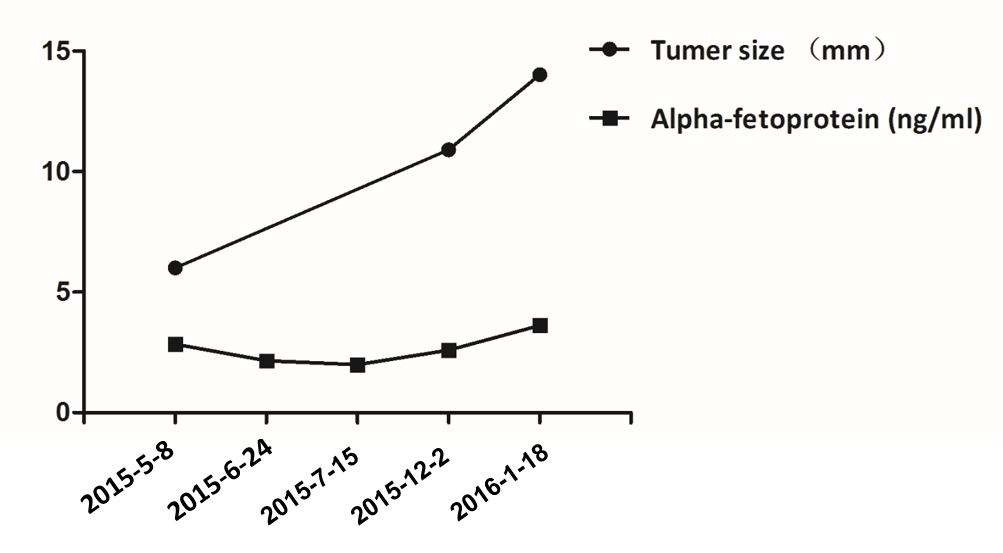

Supplement: Supplementary file 2 [file DataSheet_2.zip › Raw figure/raw image of Figure 4/level of AFP.png]

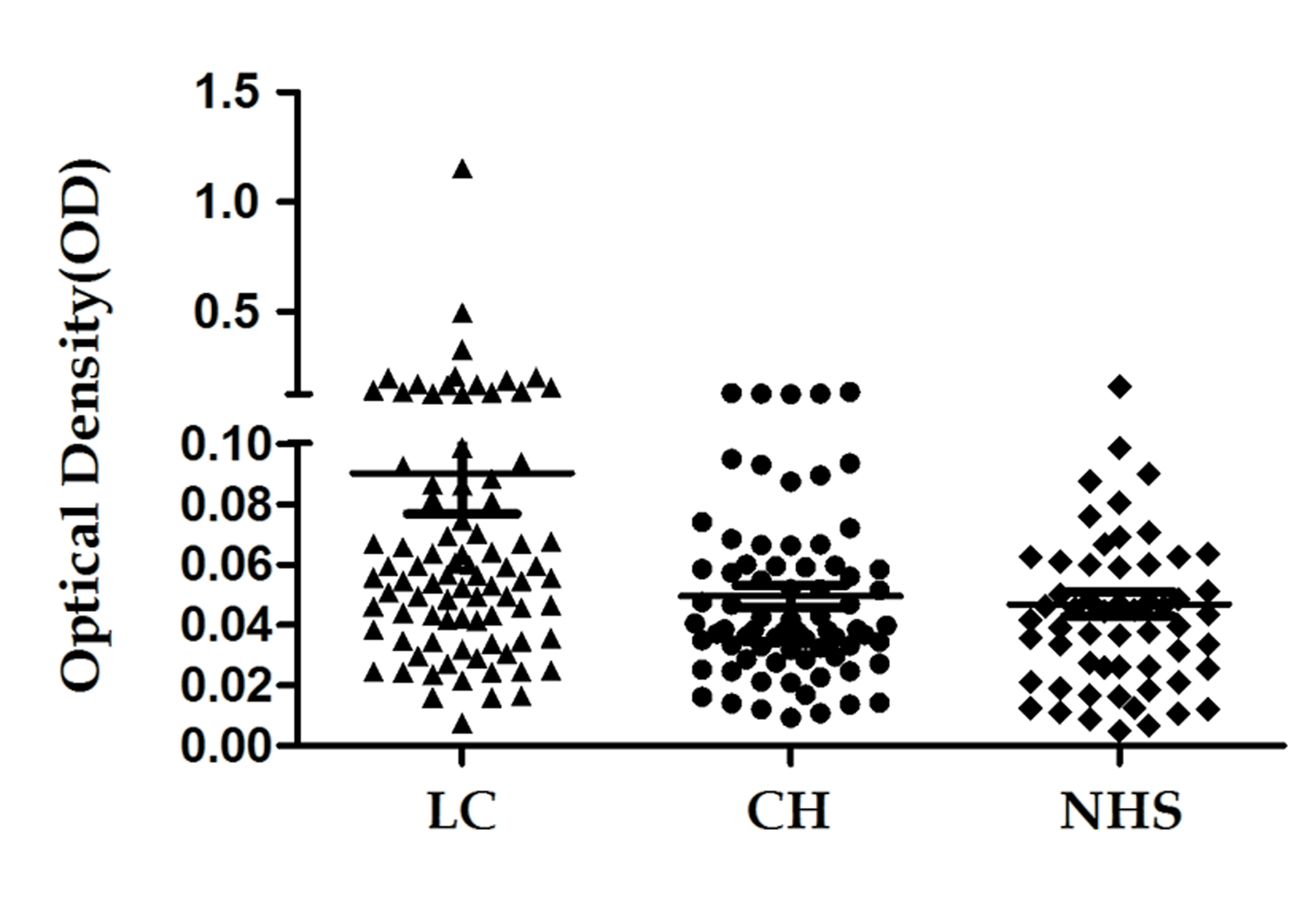

Supplement: Supplementary file 2 [file DataSheet_2.zip › Raw figure/raw image of Figure 5/FIGURE 5.tif]

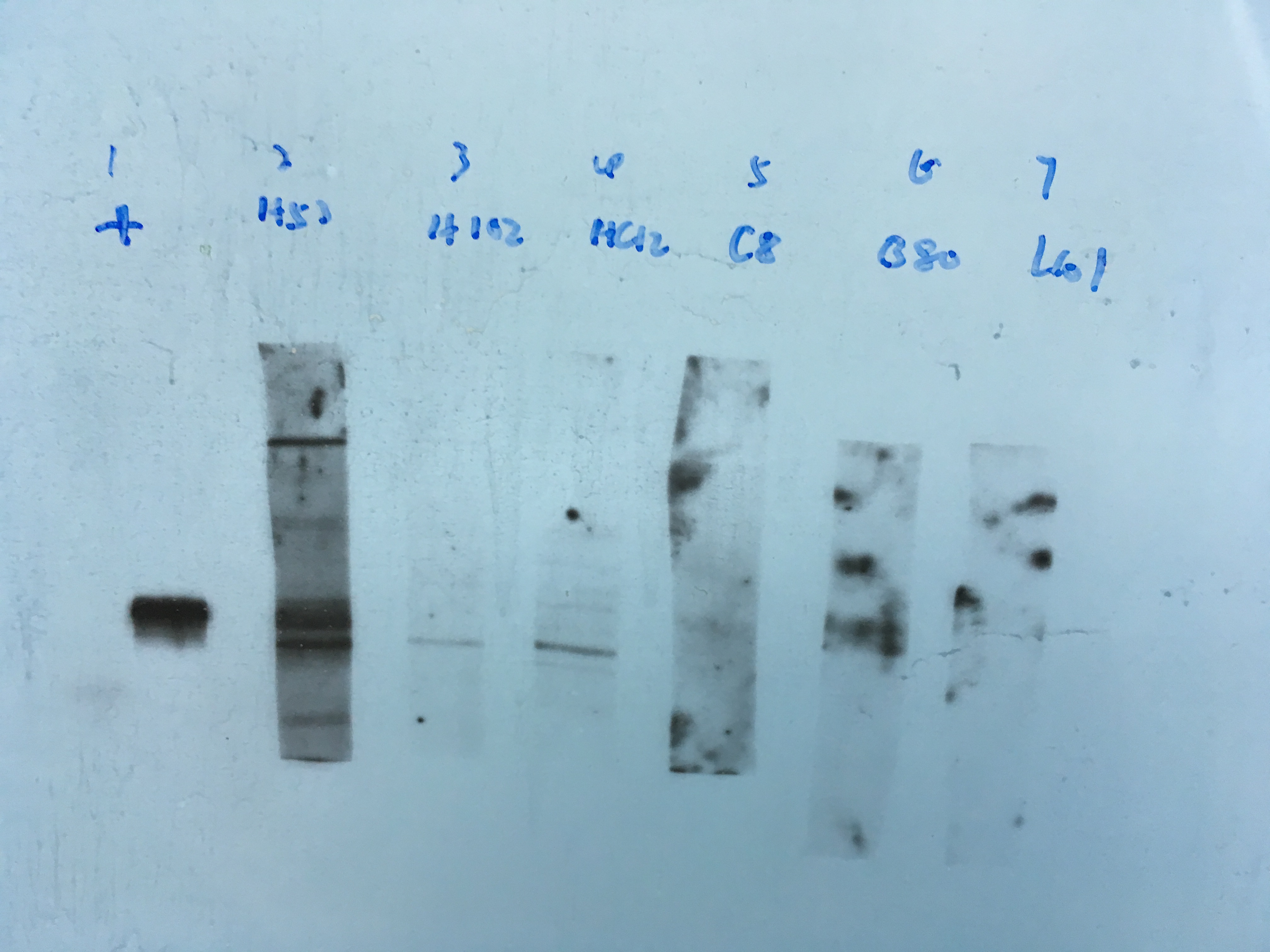

Supplement: Supplementary file 2 [file DataSheet_2.zip › Raw figure/raw image of Figure 6/WB1.JPG]

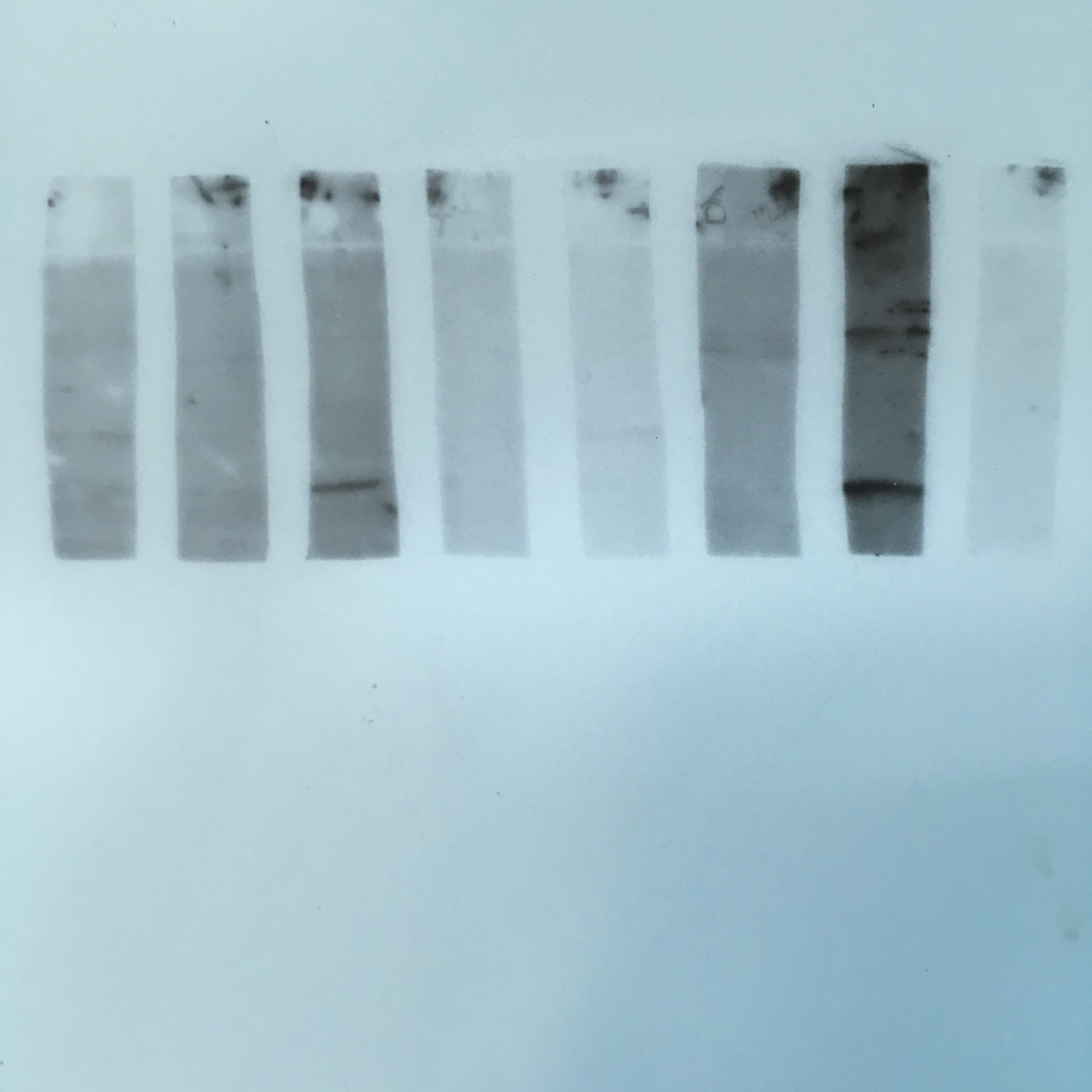

Supplement: Supplementary file 2 [file DataSheet_2.zip › Raw figure/raw image of Figure 6/WB2.JPG]

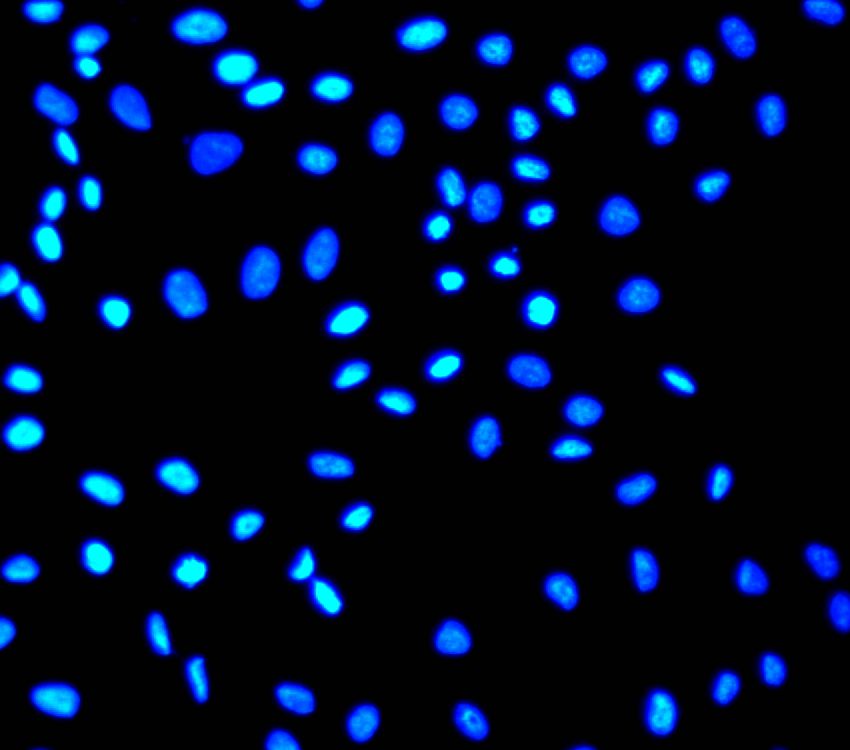

Supplement: Supplementary file 2 [file DataSheet_2.zip › Raw figure/raw image of Figure 7/LC (2)-Immunofluorescence.jpg]

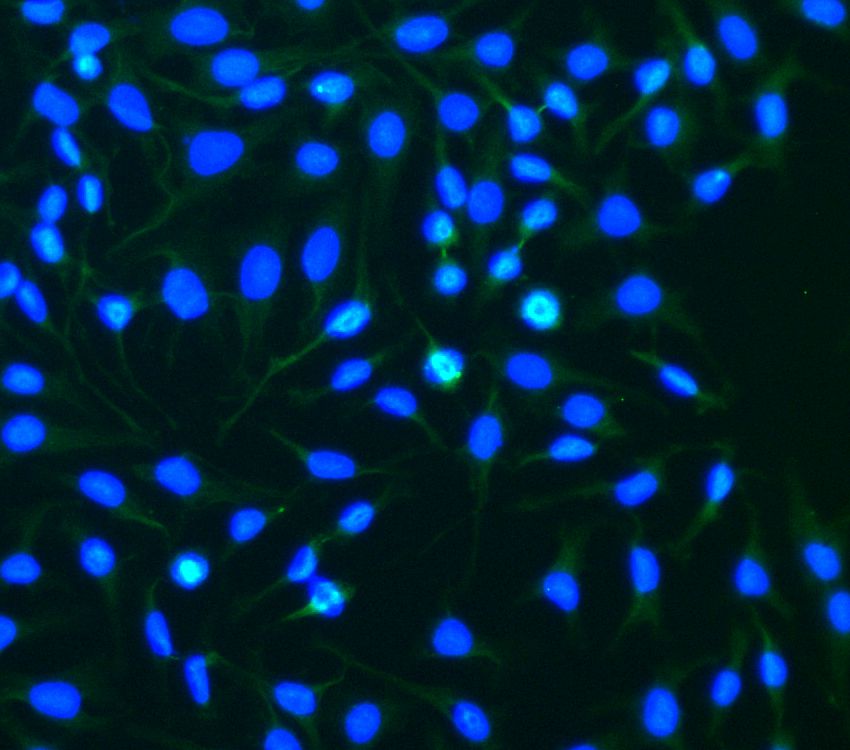

Supplement: Supplementary file 2 [file DataSheet_2.zip › Raw figure/raw image of Figure 7/LC Merged-Immunofluorescence.jpg]

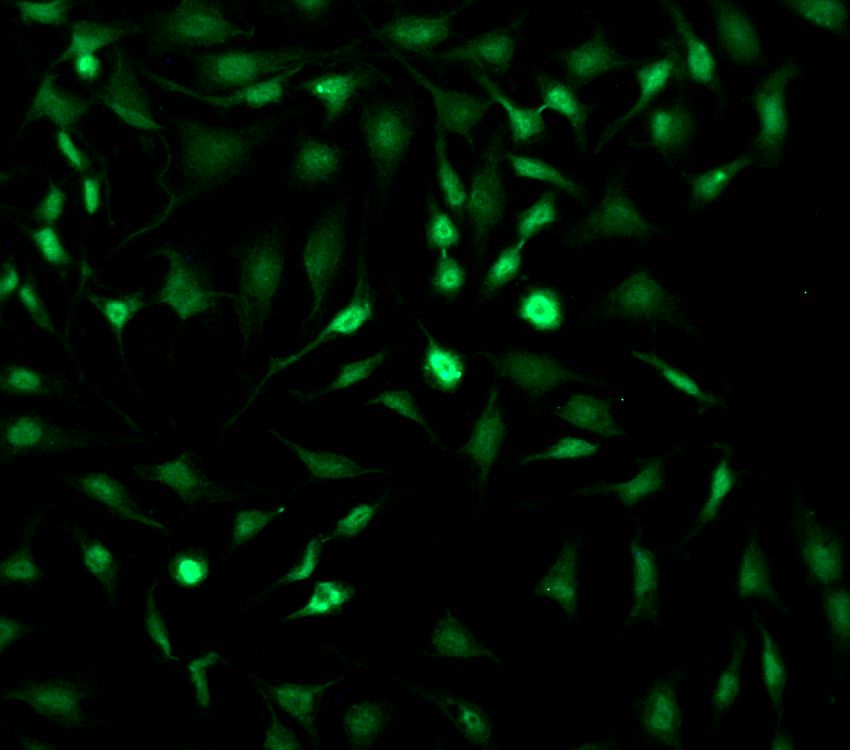

Supplement: Supplementary file 2 [file DataSheet_2.zip › Raw figure/raw image of Figure 7/LC-Immunofluorescence.jpg]

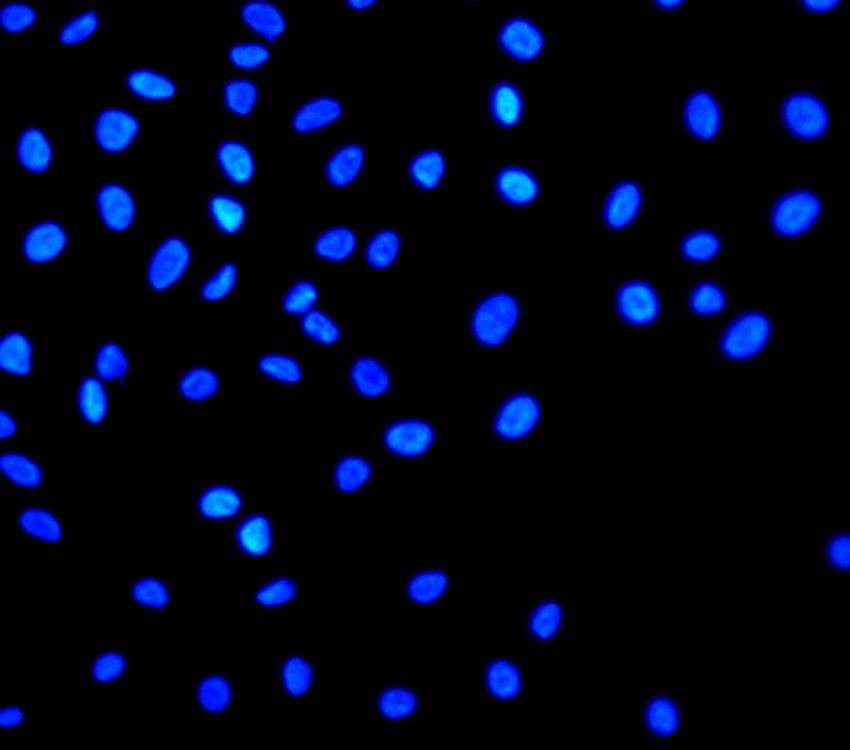

Supplement: Supplementary file 2 [file DataSheet_2.zip › Raw figure/raw image of Figure 7/NHS (2)-Immunofluorescence.jpg]

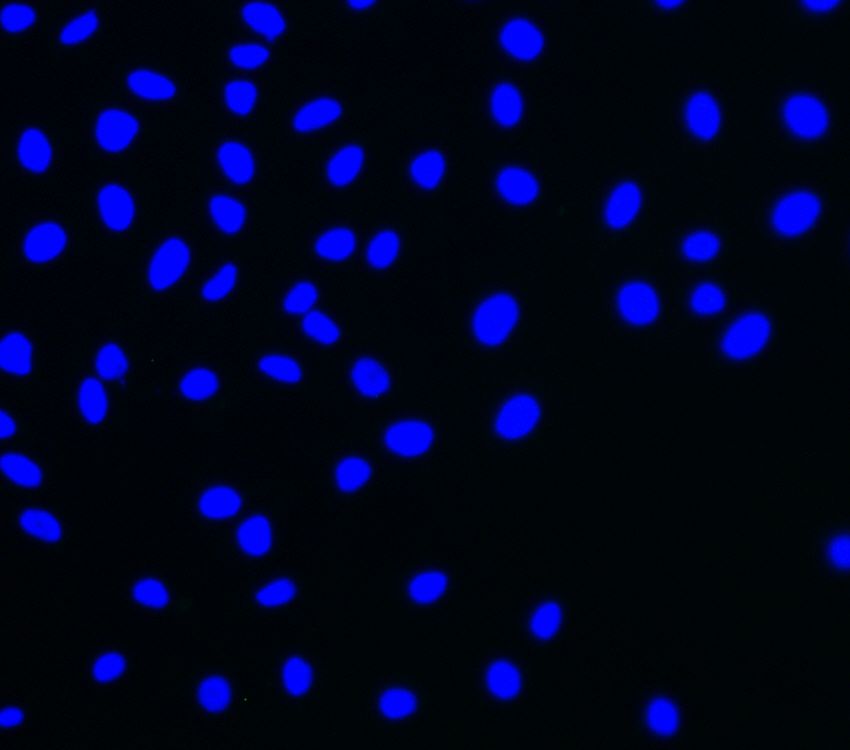

Supplement: Supplementary file 2 [file DataSheet_2.zip › Raw figure/raw image of Figure 7/NHS Merged-Immunofluorescence.jpg]

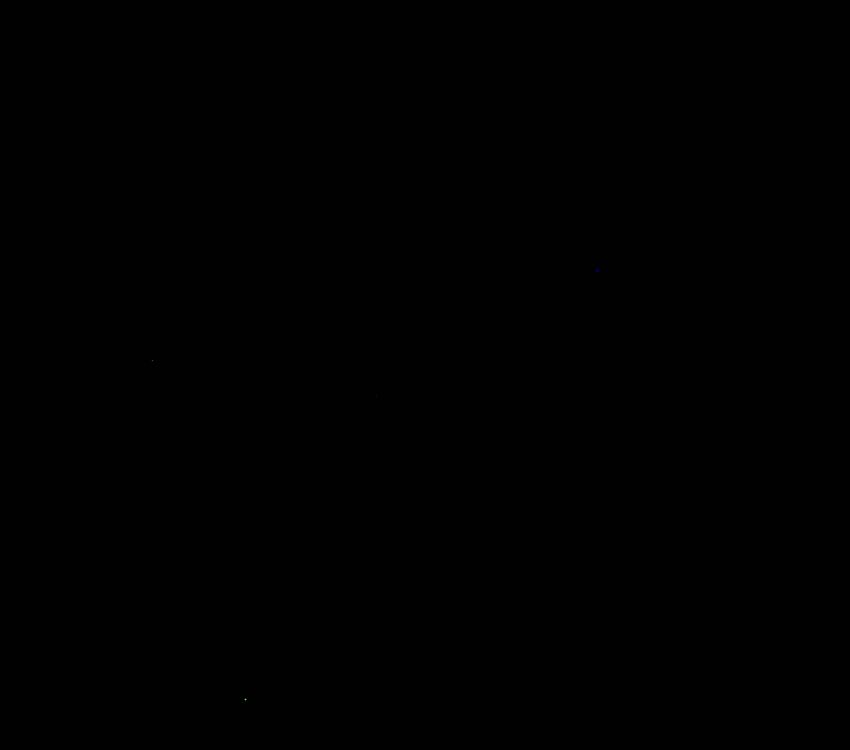

Supplement: Supplementary file 2 [file DataSheet_2.zip › Raw figure/raw image of Figure 7/NHS-Immunofluorescence.jpg]

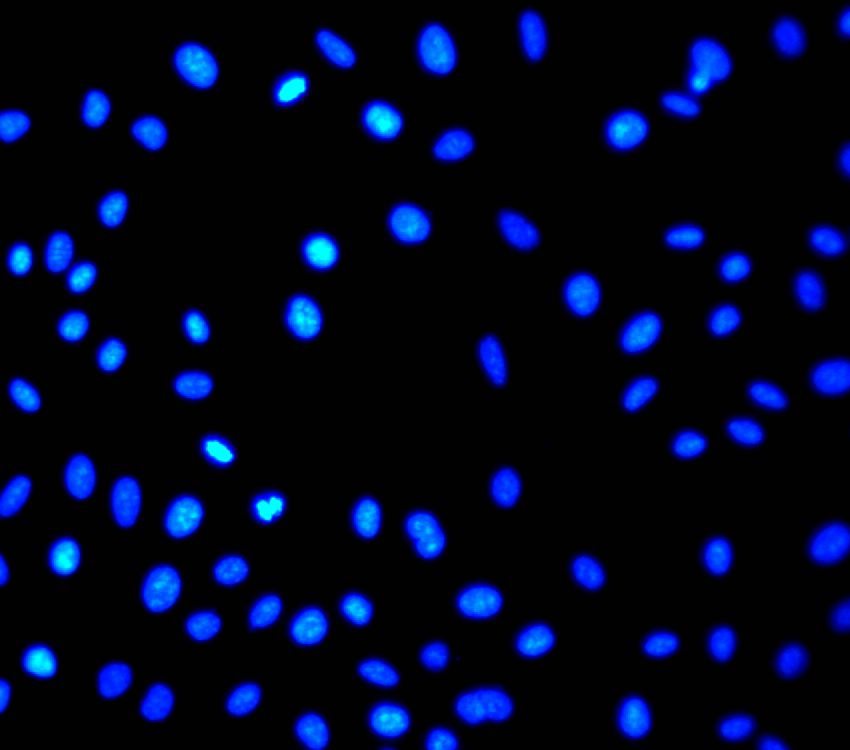

Supplement: Supplementary file 2 [file DataSheet_2.zip › Raw figure/raw image of Figure 7/positive control (2)-Immunofluorescence.jpg]

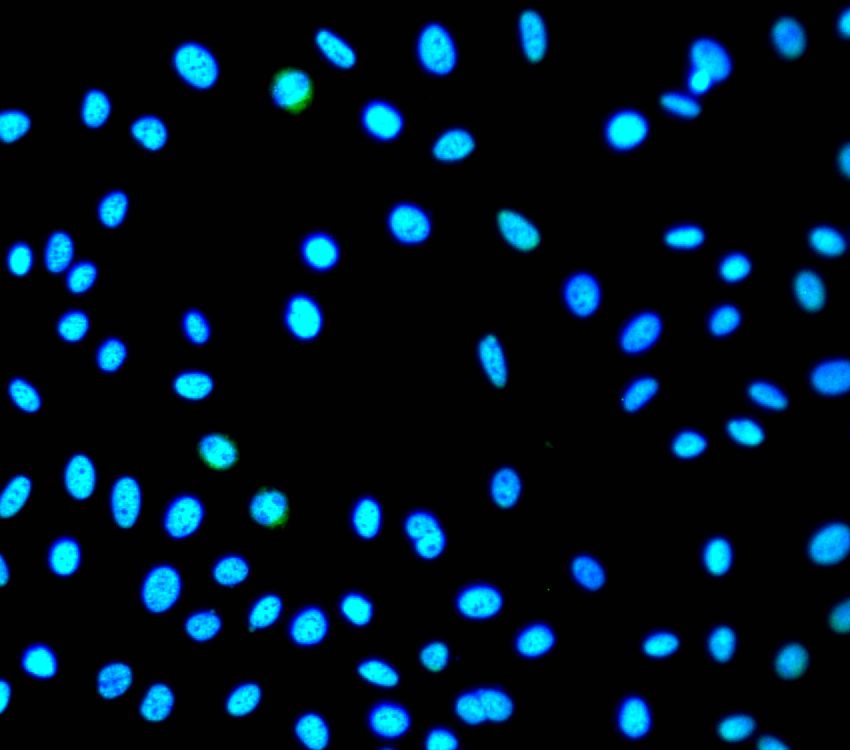

Supplement: Supplementary file 2 [file DataSheet_2.zip › Raw figure/raw image of Figure 7/positive control Merged-Immunofluorescence.jpg]

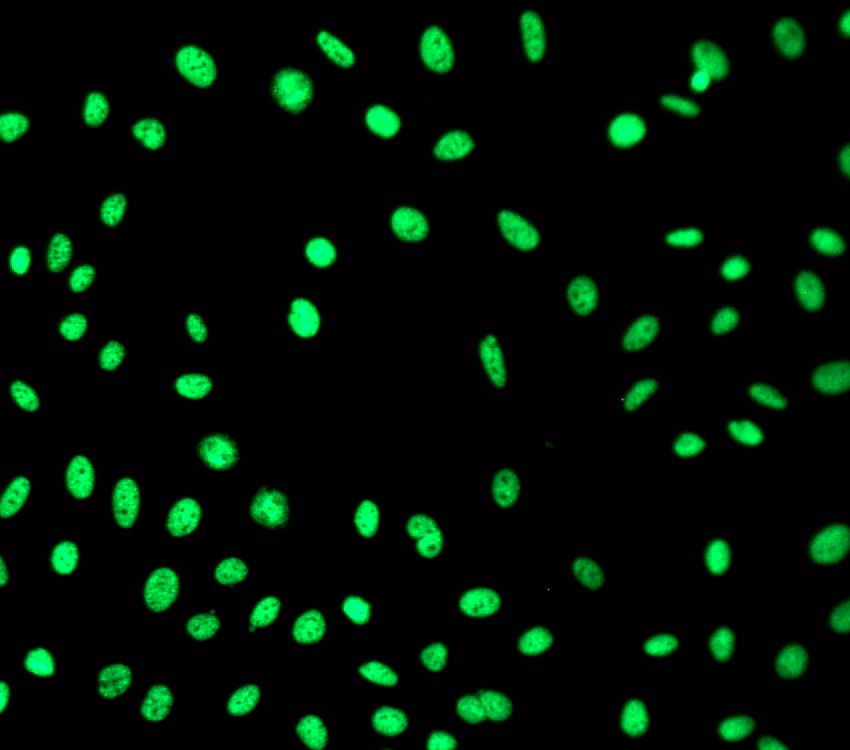

Supplement: Supplementary file 2 [file DataSheet_2.zip › Raw figure/raw image of Figure 7/positive control-Immunofluorescence.jpg]
